# Supplementary figures and images for: Ganglioside GT1b prevents selective spinal synapse removal following peripheral nerve injury (part 1 of 2)
Source: EMBO Rep. 2025 Apr 30;26(12):2994–3023. doi: 10.1038/s44319-025-00452-2 (PMC12187942; doi:10.1038/s44319-025-00452-2)

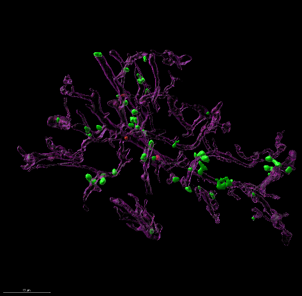

Supplement: Supplementary file 4 — Source data Fig. 1 [file 44319_2025_452_MOESM4_ESM.zip › Figure 1/Figure 1C/Astrocyte-3d.tif]

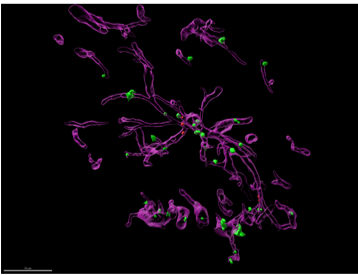

Supplement: Supplementary file 4 — Source data Fig. 1 [file 44319_2025_452_MOESM4_ESM.zip › Figure 1/Figure 1C/Astrocyte-7d.tif]

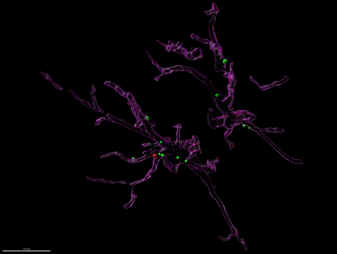

Supplement: Supplementary file 4 — Source data Fig. 1 [file 44319_2025_452_MOESM4_ESM.zip › Figure 1/Figure 1C/Astrocyte-Sham.tif]

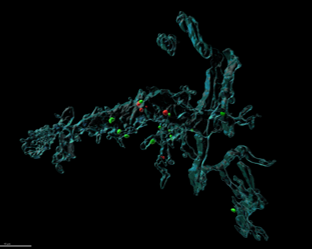

Supplement: Supplementary file 4 — Source data Fig. 1 [file 44319_2025_452_MOESM4_ESM.zip › Figure 1/Figure 1C/Microglia-3d.tif]

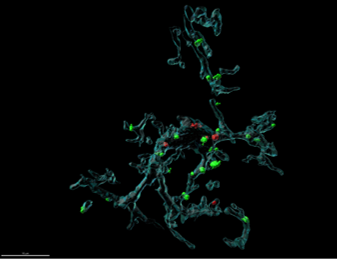

Supplement: Supplementary file 4 — Source data Fig. 1 [file 44319_2025_452_MOESM4_ESM.zip › Figure 1/Figure 1C/Microglia-7d.tif]

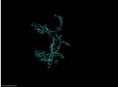

Supplement: Supplementary file 4 — Source data Fig. 1 [file 44319_2025_452_MOESM4_ESM.zip › Figure 1/Figure 1C/Microglia-Sham.tif]

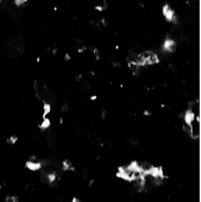

Supplement: Supplementary file 4 — Source data Fig. 1 [file 44319_2025_452_MOESM4_ESM.zip › Figure 1/Figure 1H/GT1b-3d.tif]

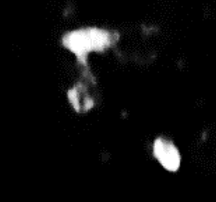

Supplement: Supplementary file 4 — Source data Fig. 1 [file 44319_2025_452_MOESM4_ESM.zip › Figure 1/Figure 1H/GT1b-7d(incorp).tif]

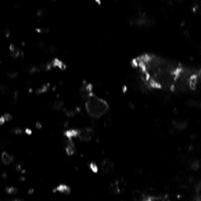

Supplement: Supplementary file 4 — Source data Fig. 1 [file 44319_2025_452_MOESM4_ESM.zip › Figure 1/Figure 1H/GT1b-7d.tif]

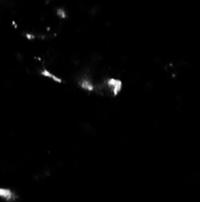

Supplement: Supplementary file 4 — Source data Fig. 1 [file 44319_2025_452_MOESM4_ESM.zip › Figure 1/Figure 1H/GT1b-Sham.tif]

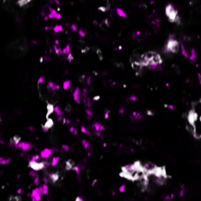

Supplement: Supplementary file 4 — Source data Fig. 1 [file 44319_2025_452_MOESM4_ESM.zip › Figure 1/Figure 1H/Merged-3d.tif]

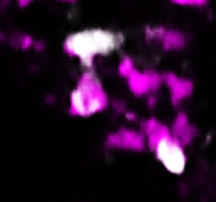

Supplement: Supplementary file 4 — Source data Fig. 1 [file 44319_2025_452_MOESM4_ESM.zip › Figure 1/Figure 1H/merged-7d(incorp).tif]

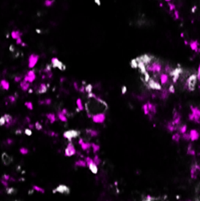

Supplement: Supplementary file 4 — Source data Fig. 1 [file 44319_2025_452_MOESM4_ESM.zip › Figure 1/Figure 1H/merged-7d.tif]

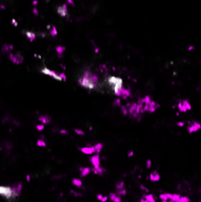

Supplement: Supplementary file 4 — Source data Fig. 1 [file 44319_2025_452_MOESM4_ESM.zip › Figure 1/Figure 1H/Merged-Sham.tif]

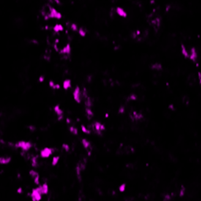

Supplement: Supplementary file 4 — Source data Fig. 1 [file 44319_2025_452_MOESM4_ESM.zip › Figure 1/Figure 1H/VGLUT2-3d.tif]

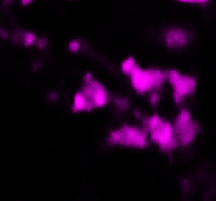

Supplement: Supplementary file 4 — Source data Fig. 1 [file 44319_2025_452_MOESM4_ESM.zip › Figure 1/Figure 1H/VGLUT2-7d(incorp).tif]

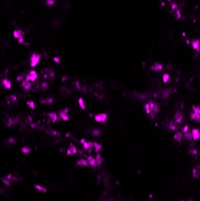

Supplement: Supplementary file 4 — Source data Fig. 1 [file 44319_2025_452_MOESM4_ESM.zip › Figure 1/Figure 1H/VGLUT2-7d.tif]

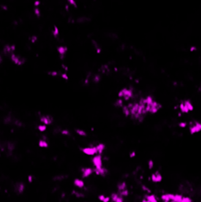

Supplement: Supplementary file 4 — Source data Fig. 1 [file 44319_2025_452_MOESM4_ESM.zip › Figure 1/Figure 1H/VGLUT2-Sham.tif]

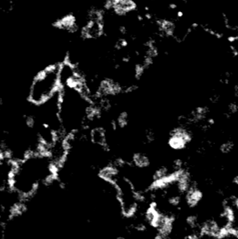

Supplement: Supplementary file 4 — Source data Fig. 1 [file 44319_2025_452_MOESM4_ESM.zip › Figure 1/Figure 1K/GT1b+ GT1b.tif]

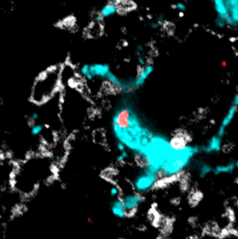

Supplement: Supplementary file 4 — Source data Fig. 1 [file 44319_2025_452_MOESM4_ESM.zip › Figure 1/Figure 1K/GT1b+ iba-1 merged.tif]

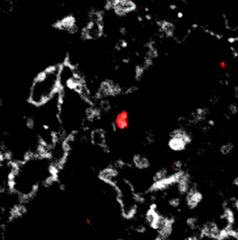

Supplement: Supplementary file 4 — Source data Fig. 1 [file 44319_2025_452_MOESM4_ESM.zip › Figure 1/Figure 1K/GT1b+ mcherry.tif]

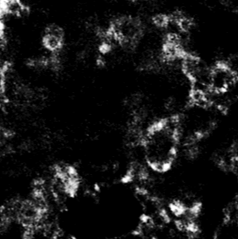

Supplement: Supplementary file 4 — Source data Fig. 1 [file 44319_2025_452_MOESM4_ESM.zip › Figure 1/Figure 1K/GT1b- GT1b.tif]

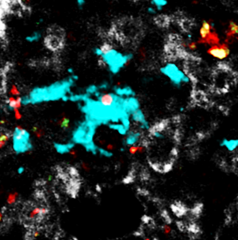

Supplement: Supplementary file 4 — Source data Fig. 1 [file 44319_2025_452_MOESM4_ESM.zip › Figure 1/Figure 1K/GT1b- iba-1 merged.tif]

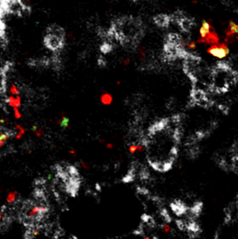

Supplement: Supplementary file 4 — Source data Fig. 1 [file 44319_2025_452_MOESM4_ESM.zip › Figure 1/Figure 1K/GT1b- mcherry.tif]

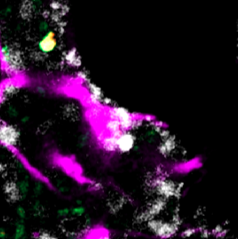

Supplement: Supplementary file 4 — Source data Fig. 1 [file 44319_2025_452_MOESM4_ESM.zip › Figure 1/Figure 1M/GT1b+ GFAP merged.tif]

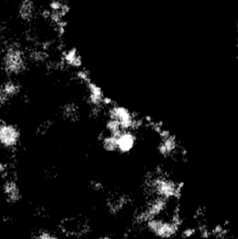

Supplement: Supplementary file 4 — Source data Fig. 1 [file 44319_2025_452_MOESM4_ESM.zip › Figure 1/Figure 1M/GT1b+ GT1b.tif]

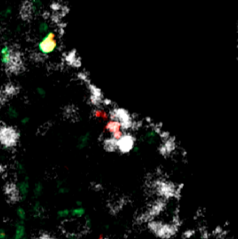

Supplement: Supplementary file 4 — Source data Fig. 1 [file 44319_2025_452_MOESM4_ESM.zip › Figure 1/Figure 1M/GT1b+ mcherry.tif]

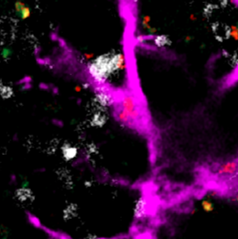

Supplement: Supplementary file 4 — Source data Fig. 1 [file 44319_2025_452_MOESM4_ESM.zip › Figure 1/Figure 1M/GT1b- GFAP merged.tif]

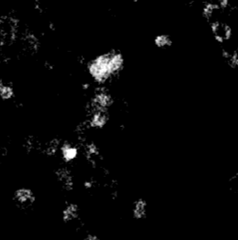

Supplement: Supplementary file 4 — Source data Fig. 1 [file 44319_2025_452_MOESM4_ESM.zip › Figure 1/Figure 1M/GT1b- GT1b.tif]

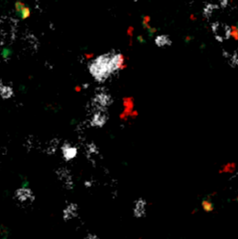

Supplement: Supplementary file 4 — Source data Fig. 1 [file 44319_2025_452_MOESM4_ESM.zip › Figure 1/Figure 1M/GT1b- mcherry.tif]

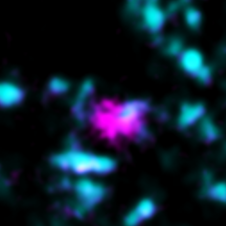

Supplement: Supplementary file 5 — Source data Fig. 2 [file 44319_2025_452_MOESM5_ESM.zip › Figure 2/Figure 2C/Phagocyotsed puncta_C3+GT1b+_C3,GT1b.tif]

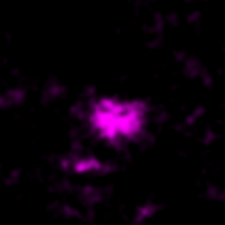

Supplement: Supplementary file 5 — Source data Fig. 2 [file 44319_2025_452_MOESM5_ESM.zip › Figure 2/Figure 2C/Phagocyotsed puncta_C3+GT1b+_C3.tif]

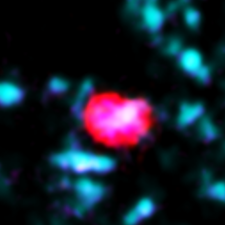

Supplement: Supplementary file 5 — Source data Fig. 2 [file 44319_2025_452_MOESM5_ESM.zip › Figure 2/Figure 2C/Phagocyotsed puncta_C3+GT1b+_ExPre.tif]

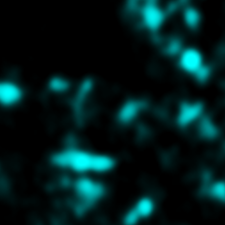

Supplement: Supplementary file 5 — Source data Fig. 2 [file 44319_2025_452_MOESM5_ESM.zip › Figure 2/Figure 2C/Phagocyotsed puncta_C3+GT1b+_GT1b.tif]

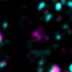

Supplement: Supplementary file 5 — Source data Fig. 2 [file 44319_2025_452_MOESM5_ESM.zip › Figure 2/Figure 2C/Phagocyotsed puncta_C3+_C3,GT1b.tif]

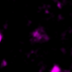

Supplement: Supplementary file 5 — Source data Fig. 2 [file 44319_2025_452_MOESM5_ESM.zip › Figure 2/Figure 2C/Phagocyotsed puncta_C3+_C3.tif]

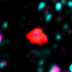

Supplement: Supplementary file 5 — Source data Fig. 2 [file 44319_2025_452_MOESM5_ESM.zip › Figure 2/Figure 2C/Phagocyotsed puncta_C3+_ExPre.tif]

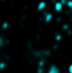

Supplement: Supplementary file 5 — Source data Fig. 2 [file 44319_2025_452_MOESM5_ESM.zip › Figure 2/Figure 2C/Phagocyotsed puncta_C3+_GT1b.tif]

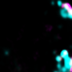

Supplement: Supplementary file 5 — Source data Fig. 2 [file 44319_2025_452_MOESM5_ESM.zip › Figure 2/Figure 2C/Phagocyotsed puncta_C3-GT1b-C3,GT1b.tif]

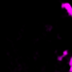

Supplement: Supplementary file 5 — Source data Fig. 2 [file 44319_2025_452_MOESM5_ESM.zip › Figure 2/Figure 2C/Phagocyotsed puncta_C3-GT1b-C3.tif]

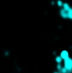

Supplement: Supplementary file 5 — Source data Fig. 2 [file 44319_2025_452_MOESM5_ESM.zip › Figure 2/Figure 2C/Phagocyotsed puncta_C3-GT1b-Gt1b.tif]

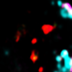

Supplement: Supplementary file 5 — Source data Fig. 2 [file 44319_2025_452_MOESM5_ESM.zip › Figure 2/Figure 2C/Phagocyotsed puncta_C3-GT1b-_ExPre.tif]

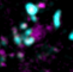

Supplement: Supplementary file 5 — Source data Fig. 2 [file 44319_2025_452_MOESM5_ESM.zip › Figure 2/Figure 2C/Synaptic puncta_C3+GT1b+_C3,GT1b.tif]

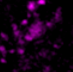

Supplement: Supplementary file 5 — Source data Fig. 2 [file 44319_2025_452_MOESM5_ESM.zip › Figure 2/Figure 2C/Synaptic puncta_C3+GT1b+_C3.tif]

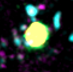

Supplement: Supplementary file 5 — Source data Fig. 2 [file 44319_2025_452_MOESM5_ESM.zip › Figure 2/Figure 2C/Synaptic puncta_C3+GT1b+_ExPre.tif]

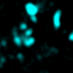

Supplement: Supplementary file 5 — Source data Fig. 2 [file 44319_2025_452_MOESM5_ESM.zip › Figure 2/Figure 2C/Synaptic puncta_C3+GT1b+_GT1b.tif]

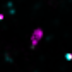

Supplement: Supplementary file 5 — Source data Fig. 2 [file 44319_2025_452_MOESM5_ESM.zip › Figure 2/Figure 2C/Synaptic puncta_C3+_C3,GT1b.tif]

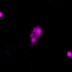

Supplement: Supplementary file 5 — Source data Fig. 2 [file 44319_2025_452_MOESM5_ESM.zip › Figure 2/Figure 2C/Synaptic puncta_C3+_C3.tif]

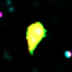

Supplement: Supplementary file 5 — Source data Fig. 2 [file 44319_2025_452_MOESM5_ESM.zip › Figure 2/Figure 2C/Synaptic puncta_C3+_ExPre.tif]

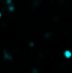

Supplement: Supplementary file 5 — Source data Fig. 2 [file 44319_2025_452_MOESM5_ESM.zip › Figure 2/Figure 2C/Synaptic puncta_C3+_GT1b.tif]

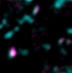

Supplement: Supplementary file 5 — Source data Fig. 2 [file 44319_2025_452_MOESM5_ESM.zip › Figure 2/Figure 2C/Synaptic puncta_C3-GT1b-_C3,GT1b.tif]

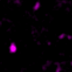

Supplement: Supplementary file 5 — Source data Fig. 2 [file 44319_2025_452_MOESM5_ESM.zip › Figure 2/Figure 2C/Synaptic puncta_C3-GT1b-_C3.tif]

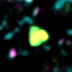

Supplement: Supplementary file 5 — Source data Fig. 2 [file 44319_2025_452_MOESM5_ESM.zip › Figure 2/Figure 2C/Synaptic puncta_C3-GT1b-_ExPre.tif]

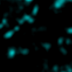

Supplement: Supplementary file 5 — Source data Fig. 2 [file 44319_2025_452_MOESM5_ESM.zip › Figure 2/Figure 2C/Synaptic puncta_C3-GT1b-_GT1b.tif]

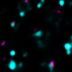

Supplement: Supplementary file 5 — Source data Fig. 2 [file 44319_2025_452_MOESM5_ESM.zip › Figure 2/Figure 2C/Synaptic puncta_GT1b+_C3,GT1b.tif]

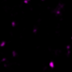

Supplement: Supplementary file 5 — Source data Fig. 2 [file 44319_2025_452_MOESM5_ESM.zip › Figure 2/Figure 2C/Synaptic puncta_GT1b+_C3.tif]

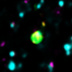

Supplement: Supplementary file 5 — Source data Fig. 2 [file 44319_2025_452_MOESM5_ESM.zip › Figure 2/Figure 2C/Synaptic puncta_GT1b+_ExPre.tif]

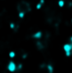

Supplement: Supplementary file 5 — Source data Fig. 2 [file 44319_2025_452_MOESM5_ESM.zip › Figure 2/Figure 2C/Synaptic puncta_GT1b+_GT1b.tif]

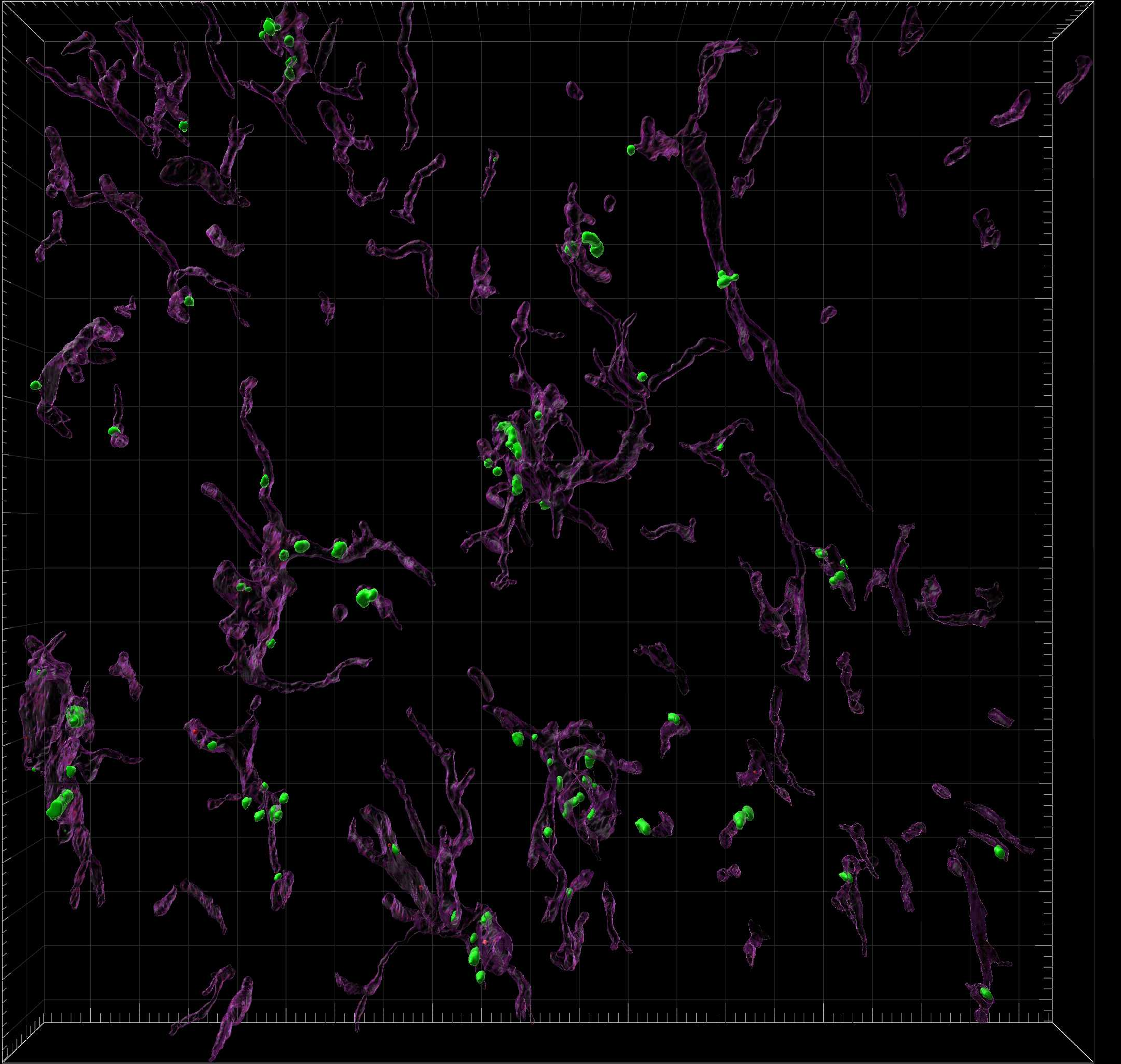

Supplement: Supplementary file 6 — Source data Fig. 3 [file 44319_2025_452_MOESM6_ESM.zip › Figure 3/Figure 3B/PDMP-GFAP.tif]

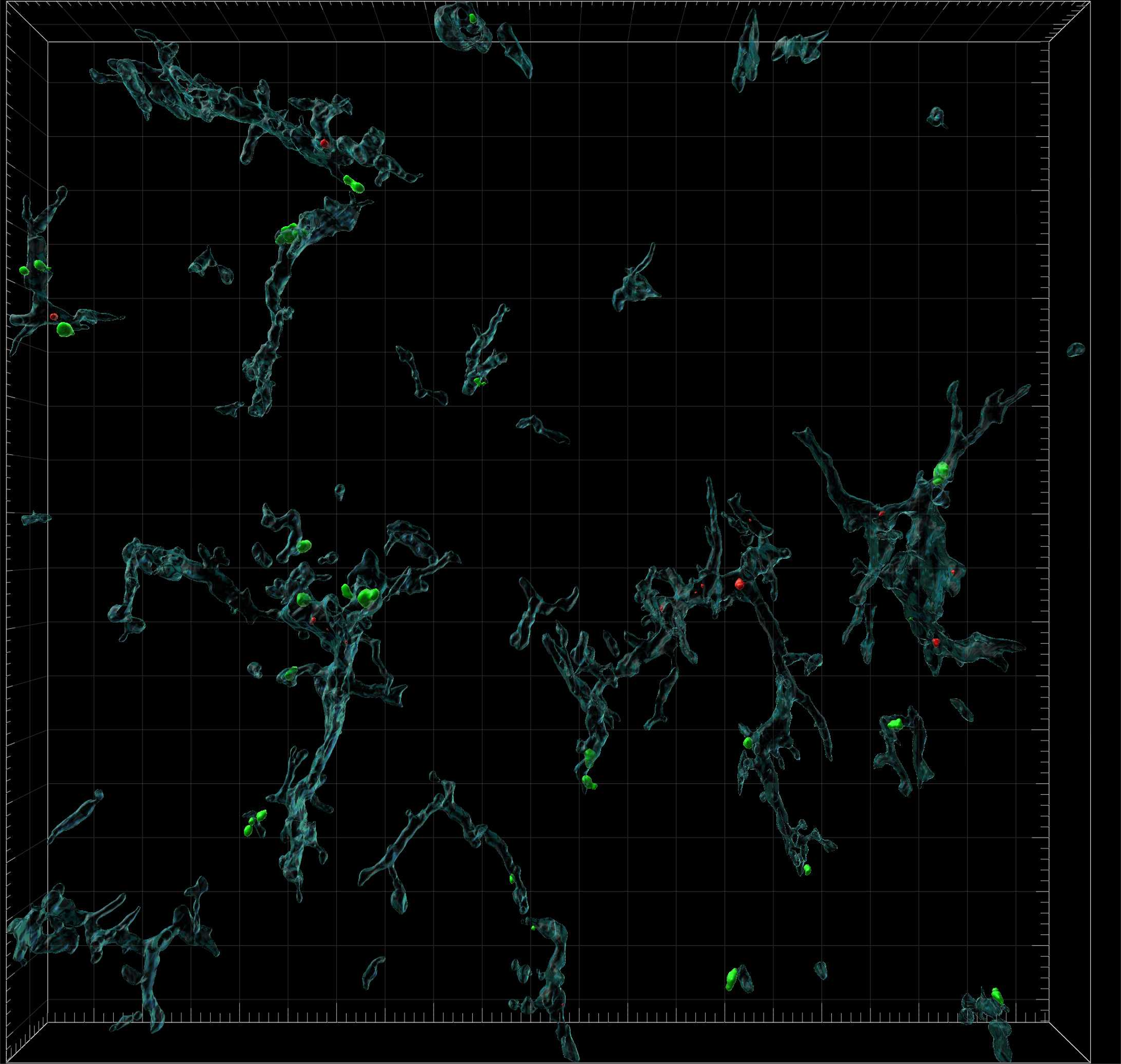

Supplement: Supplementary file 6 — Source data Fig. 3 [file 44319_2025_452_MOESM6_ESM.zip › Figure 3/Figure 3B/PDMP-Iba-1.tif]

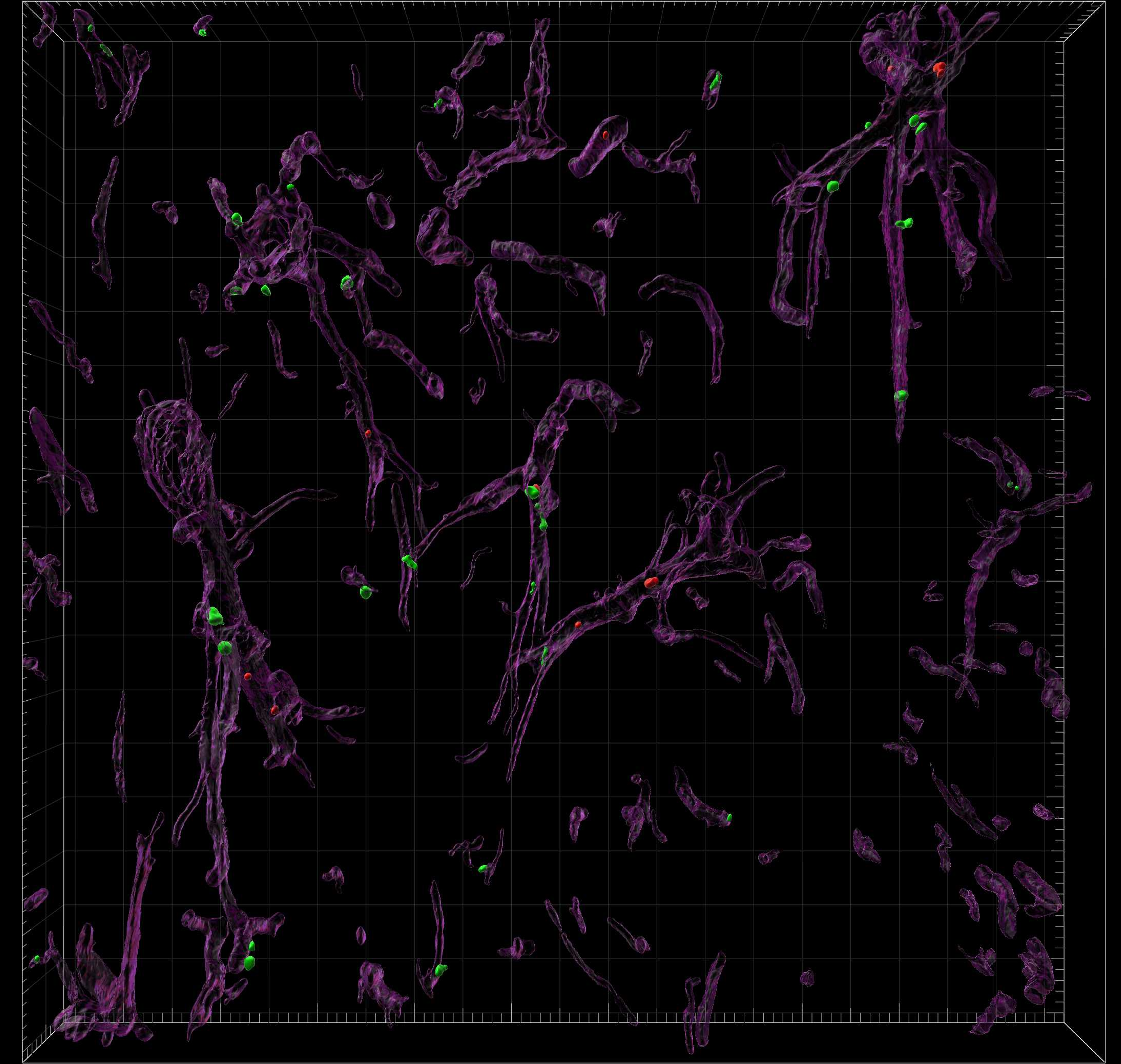

Supplement: Supplementary file 6 — Source data Fig. 3 [file 44319_2025_452_MOESM6_ESM.zip › Figure 3/Figure 3B/Veh-GFAP.tif]

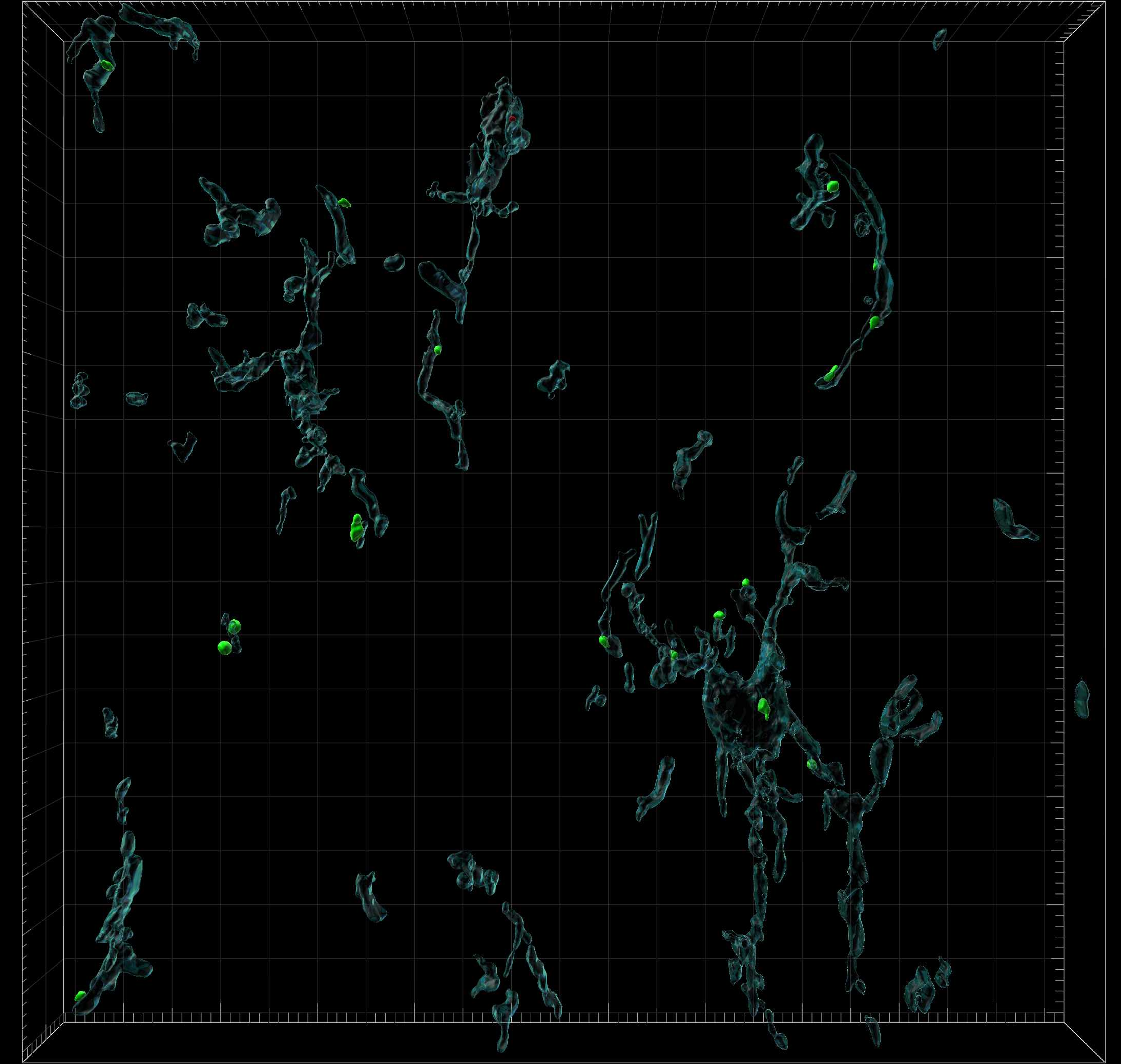

Supplement: Supplementary file 6 — Source data Fig. 3 [file 44319_2025_452_MOESM6_ESM.zip › Figure 3/Figure 3B/Veh-Iba-1.tif]

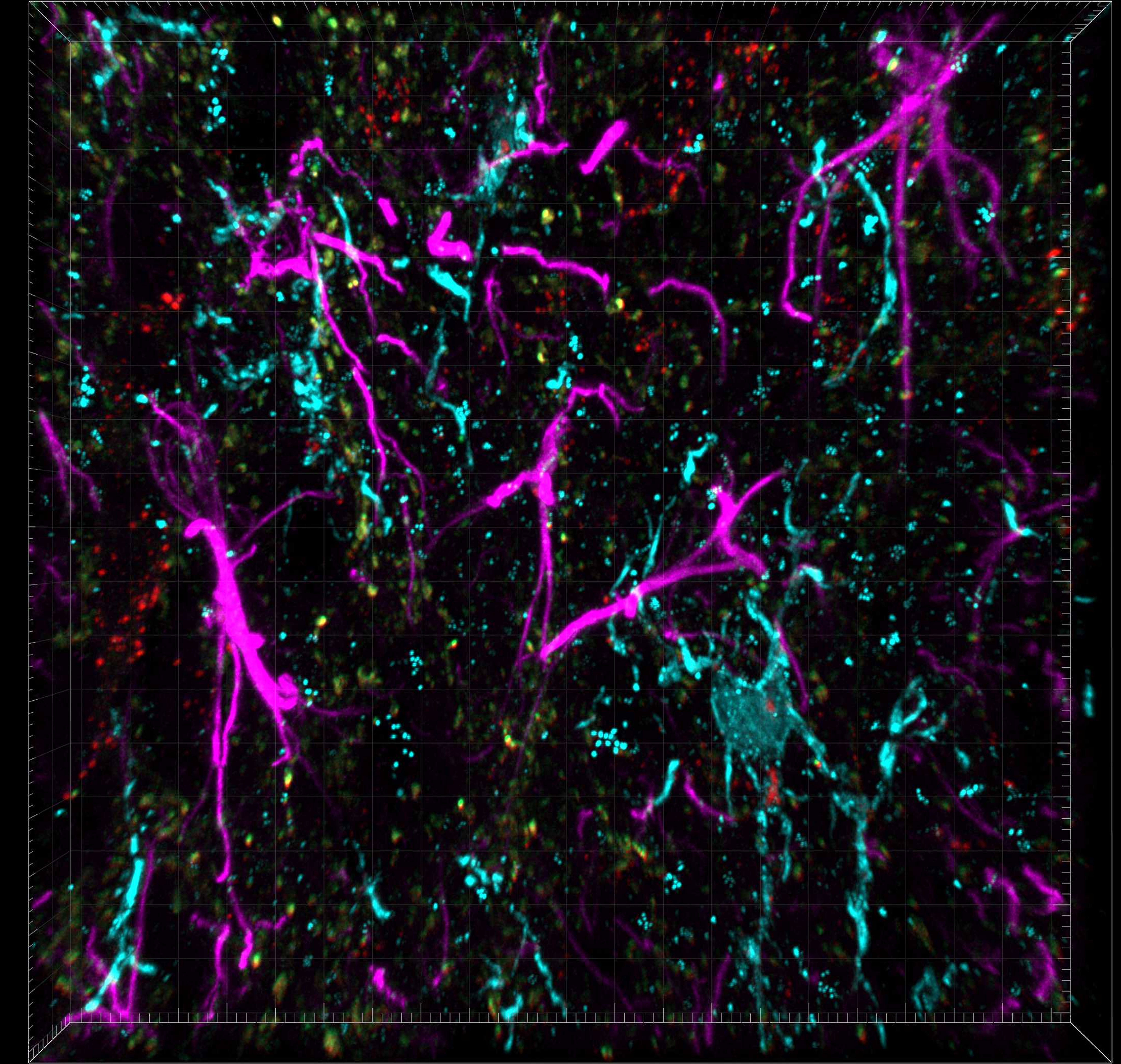

Supplement: Supplementary file 6 — Source data Fig. 3 [file 44319_2025_452_MOESM6_ESM.zip › Figure 3/Figure 3B/Veh-merged.tif]

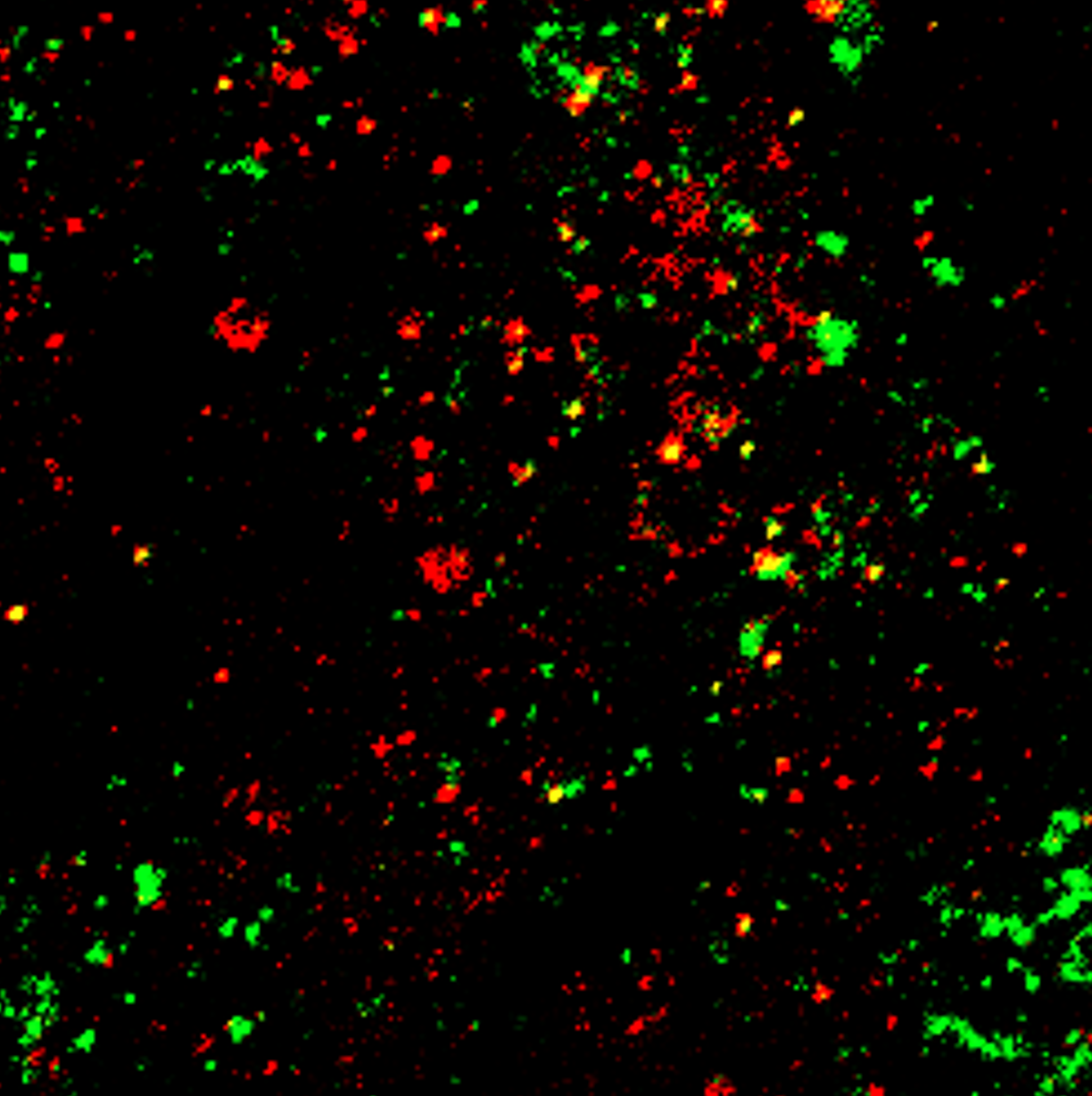

Supplement: Supplementary file 6 — Source data Fig. 3 [file 44319_2025_452_MOESM6_ESM.zip › Figure 3/Figure 3D/PDMP-3d_Cont.tif]

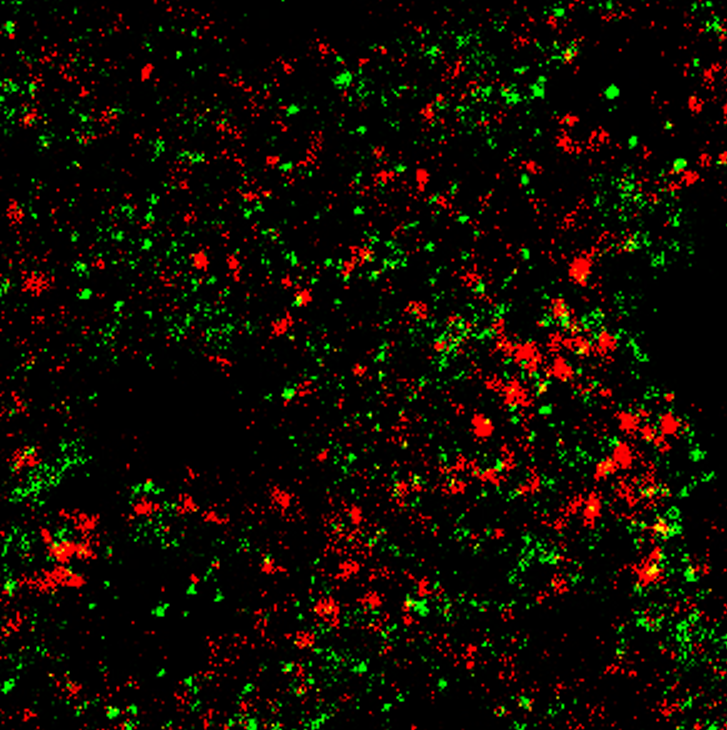

Supplement: Supplementary file 6 — Source data Fig. 3 [file 44319_2025_452_MOESM6_ESM.zip › Figure 3/Figure 3D/PDMP-3d_ipsi.tif]

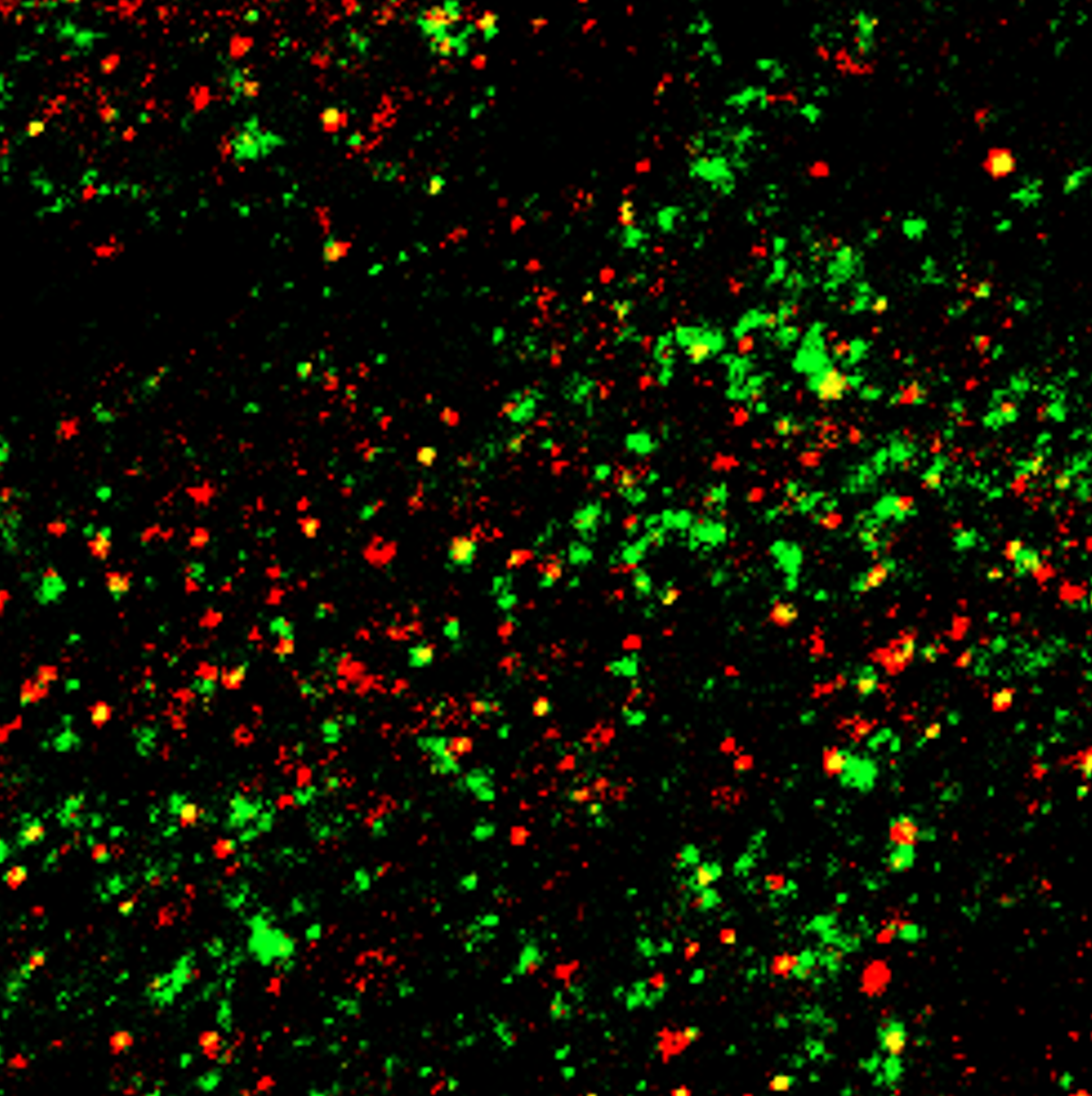

Supplement: Supplementary file 6 — Source data Fig. 3 [file 44319_2025_452_MOESM6_ESM.zip › Figure 3/Figure 3D/PDMP-Sham_Cont.tif]

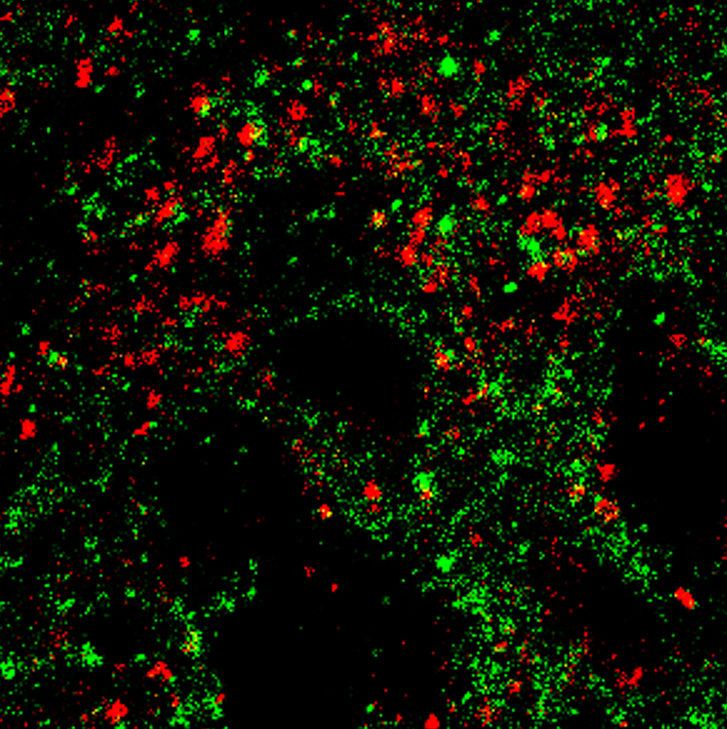

Supplement: Supplementary file 6 — Source data Fig. 3 [file 44319_2025_452_MOESM6_ESM.zip › Figure 3/Figure 3D/PDMP-Sham_ipsi.tif]

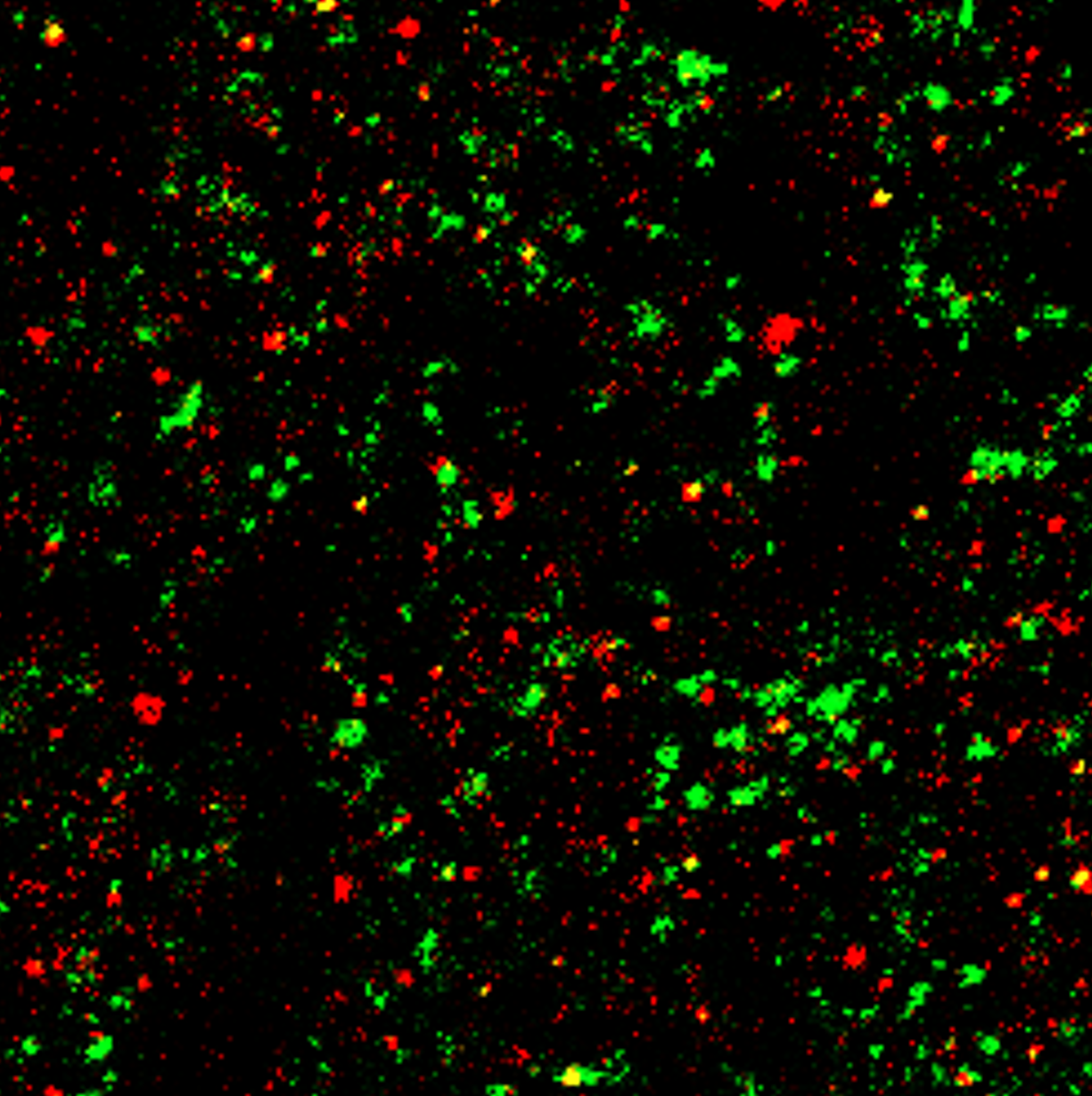

Supplement: Supplementary file 6 — Source data Fig. 3 [file 44319_2025_452_MOESM6_ESM.zip › Figure 3/Figure 3D/Veh-3d_Cont.tif]

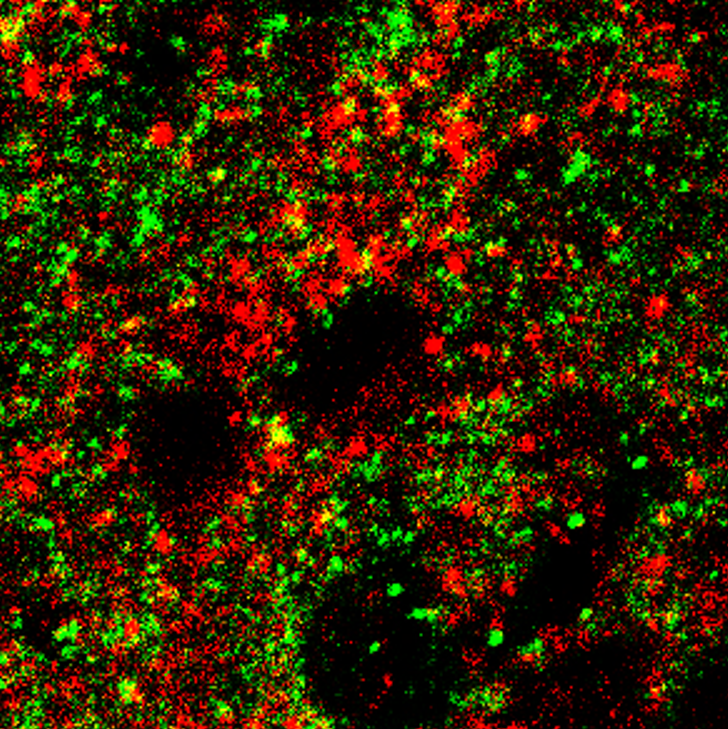

Supplement: Supplementary file 6 — Source data Fig. 3 [file 44319_2025_452_MOESM6_ESM.zip › Figure 3/Figure 3D/Veh-3d_ipsi.tif]

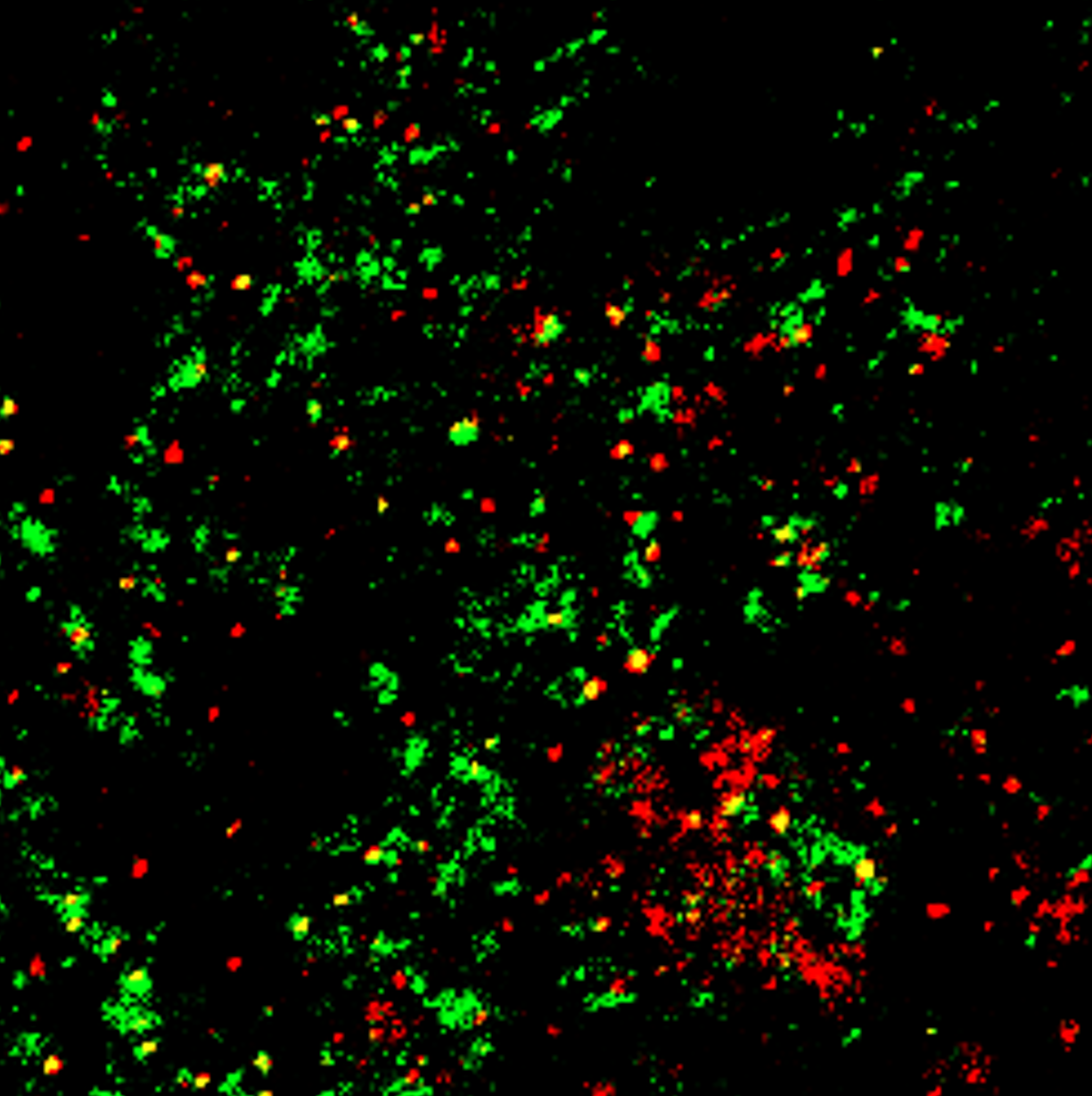

Supplement: Supplementary file 6 — Source data Fig. 3 [file 44319_2025_452_MOESM6_ESM.zip › Figure 3/Figure 3D/Veh-Sham_Cont.tif]

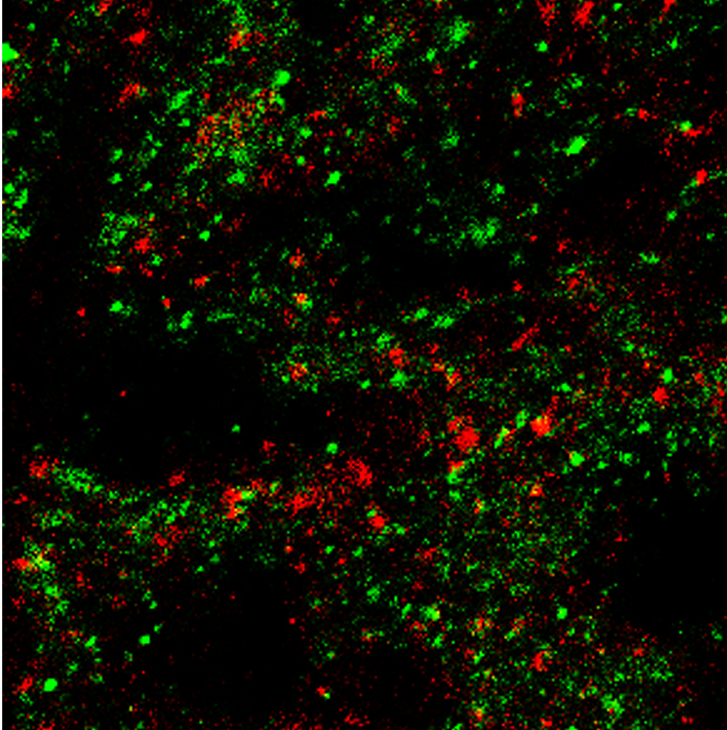

Supplement: Supplementary file 6 — Source data Fig. 3 [file 44319_2025_452_MOESM6_ESM.zip › Figure 3/Figure 3D/Veh-Sham_ipsi.tif]

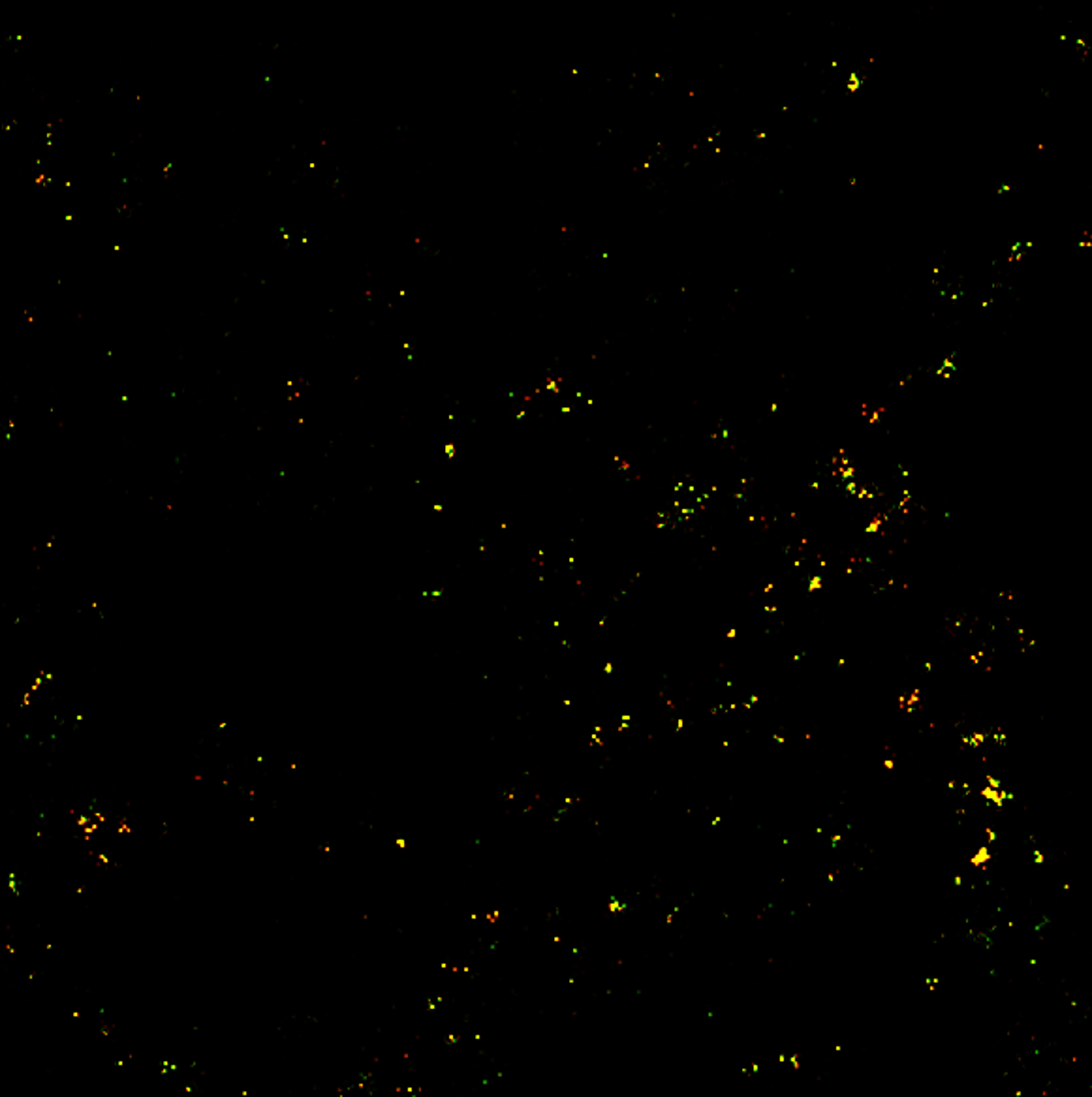

Supplement: Supplementary file 6 — Source data Fig. 3 [file 44319_2025_452_MOESM6_ESM.zip › Figure 3/Figure 3E/PDMP-3d merged.tif]

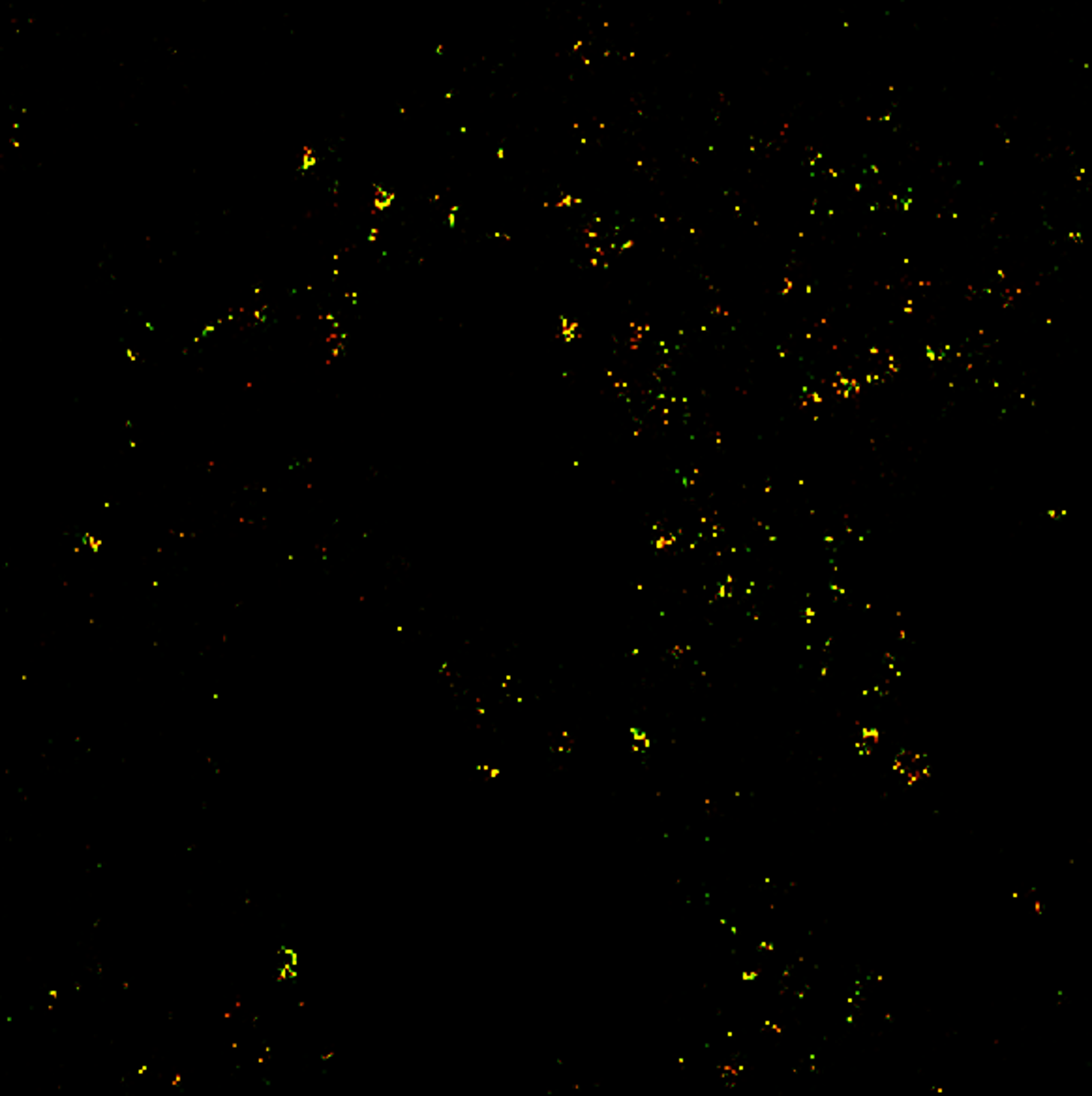

Supplement: Supplementary file 6 — Source data Fig. 3 [file 44319_2025_452_MOESM6_ESM.zip › Figure 3/Figure 3E/PDMP-Sham merged.tif]

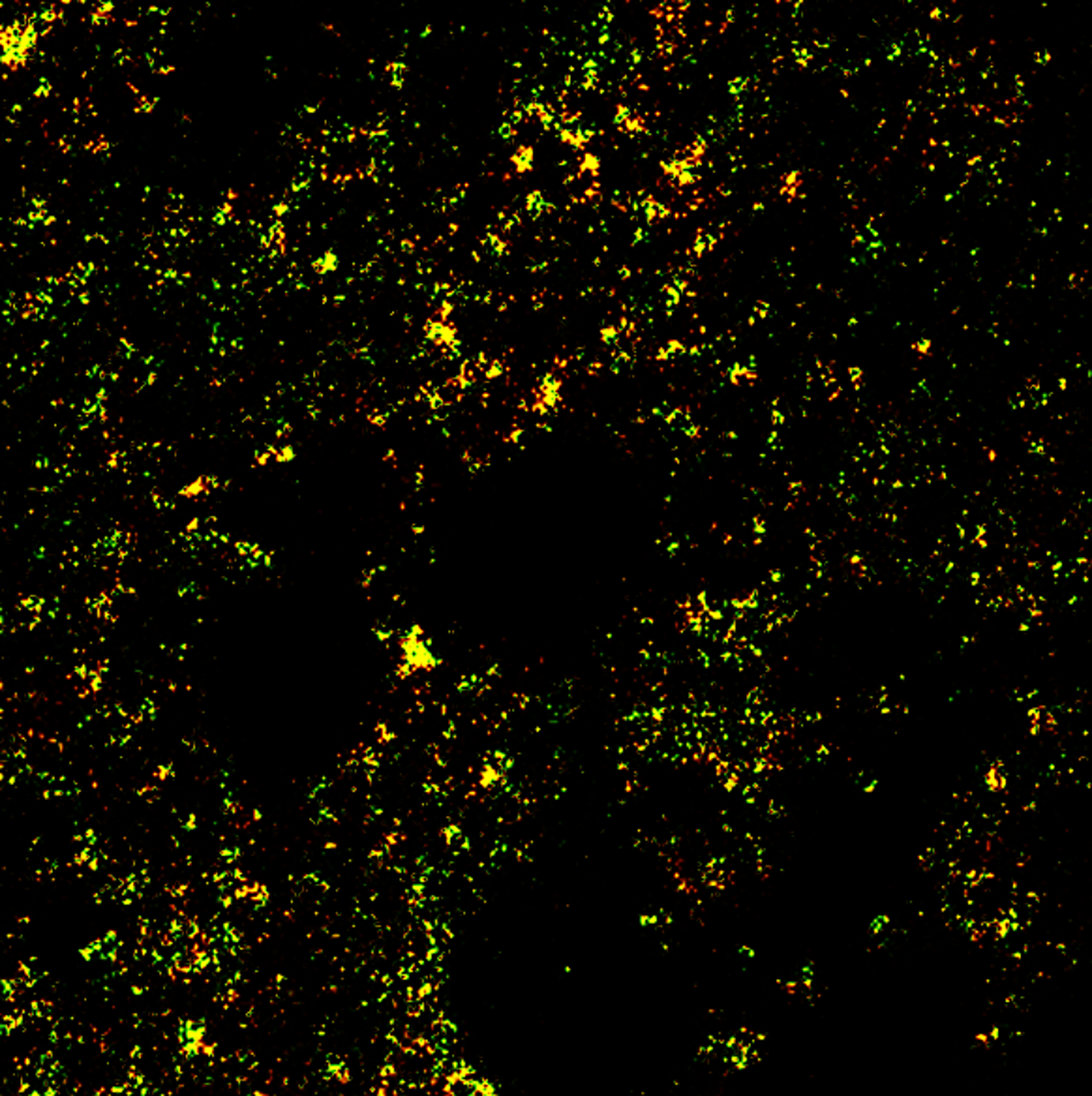

Supplement: Supplementary file 6 — Source data Fig. 3 [file 44319_2025_452_MOESM6_ESM.zip › Figure 3/Figure 3E/Veh-3d merged.tif]

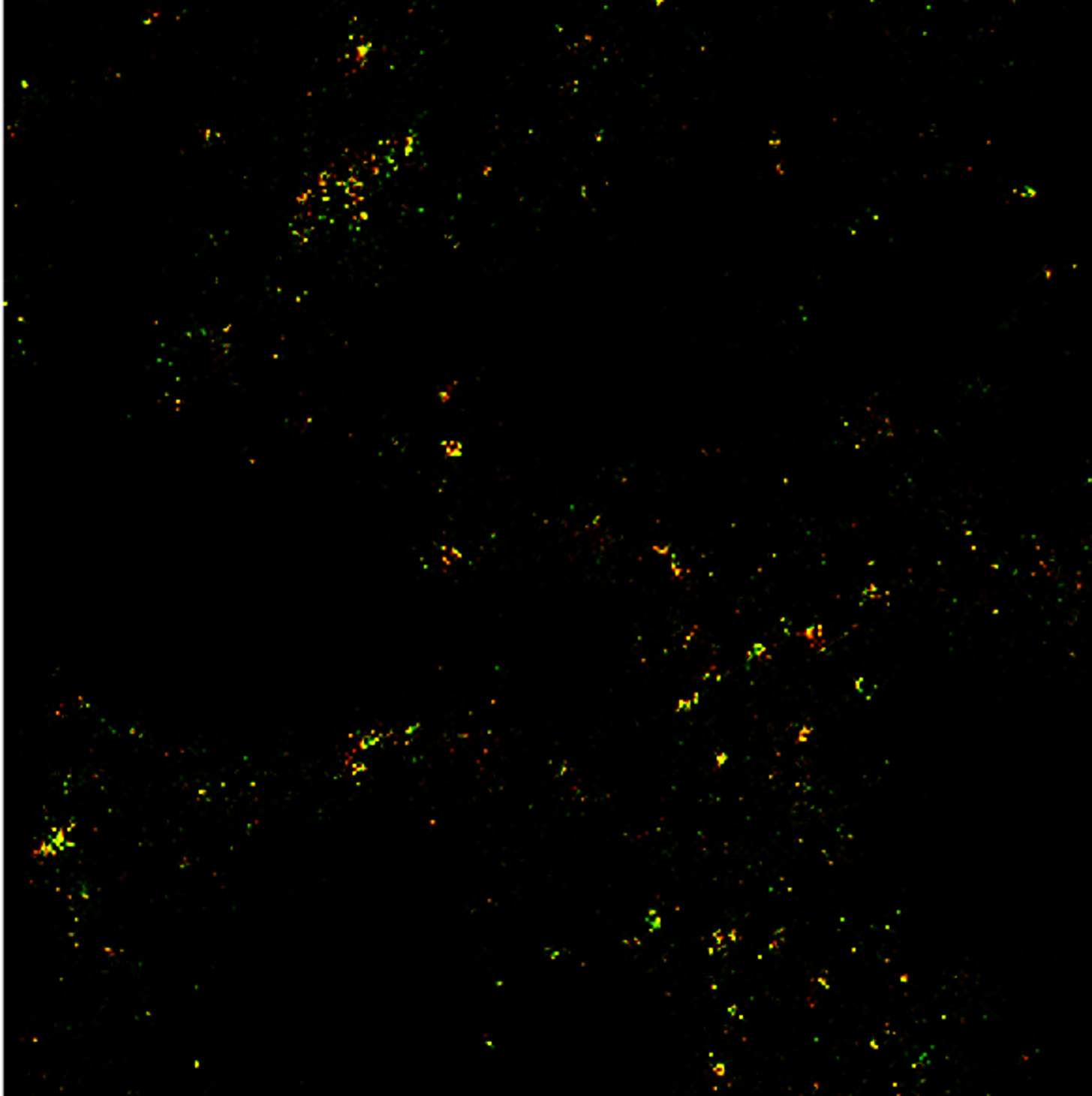

Supplement: Supplementary file 6 — Source data Fig. 3 [file 44319_2025_452_MOESM6_ESM.zip › Figure 3/Figure 3E/Veh-Sham merged.tif]

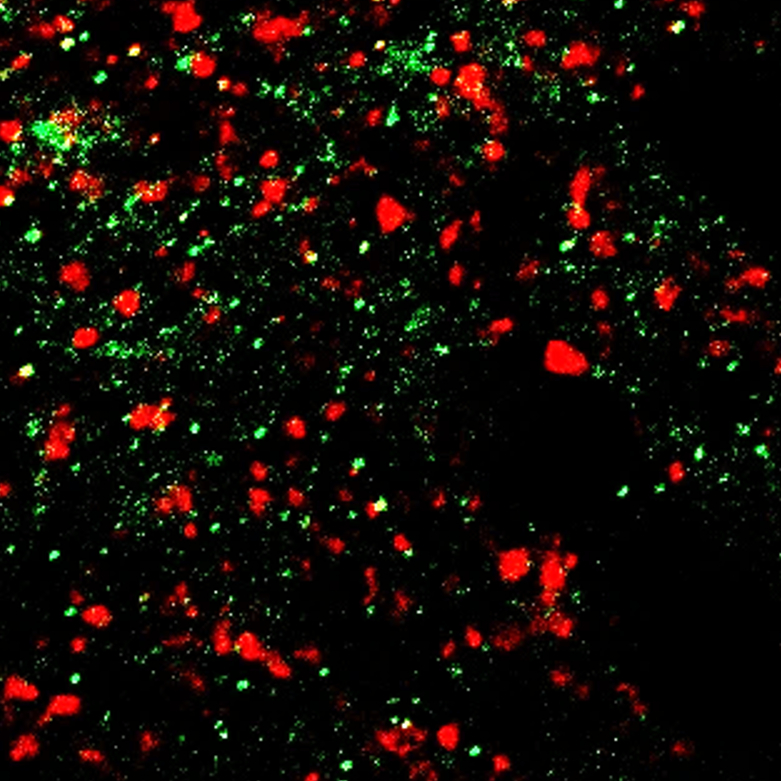

Supplement: Supplementary file 6 — Source data Fig. 3 [file 44319_2025_452_MOESM6_ESM.zip › Figure 3/Figure 3J/St3gal2,3 dKO_3d_cont.tif]

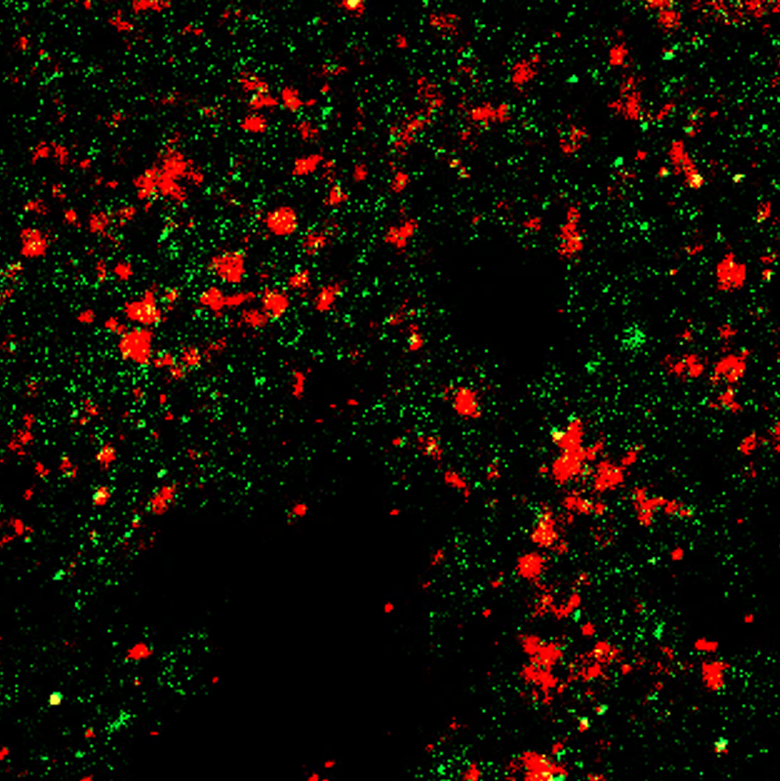

Supplement: Supplementary file 6 — Source data Fig. 3 [file 44319_2025_452_MOESM6_ESM.zip › Figure 3/Figure 3J/St3gal2,3 dKO_3d_ipsi.tif]

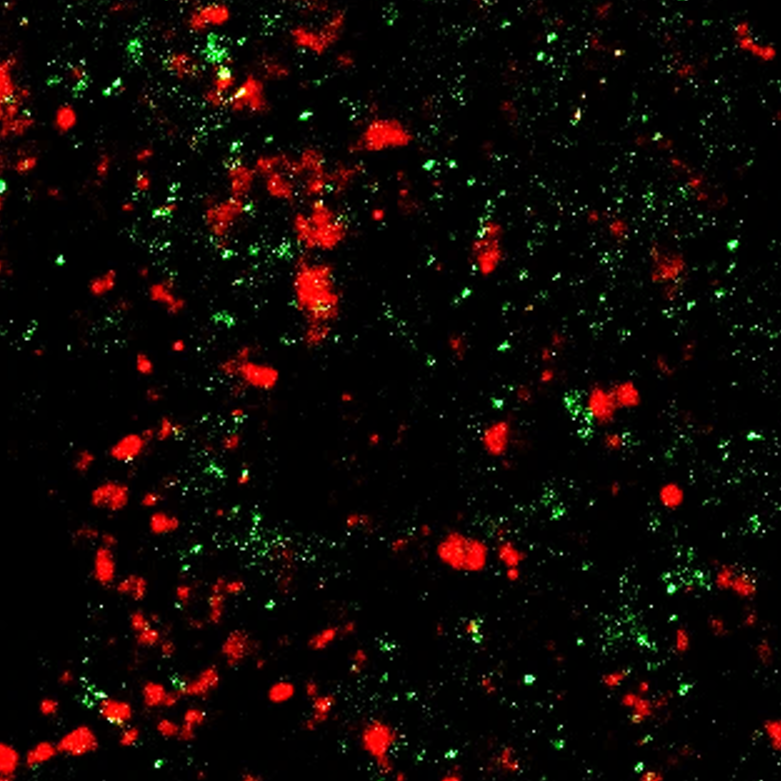

Supplement: Supplementary file 6 — Source data Fig. 3 [file 44319_2025_452_MOESM6_ESM.zip › Figure 3/Figure 3J/St3gal2,3 dKO_Sham_cont.tif]

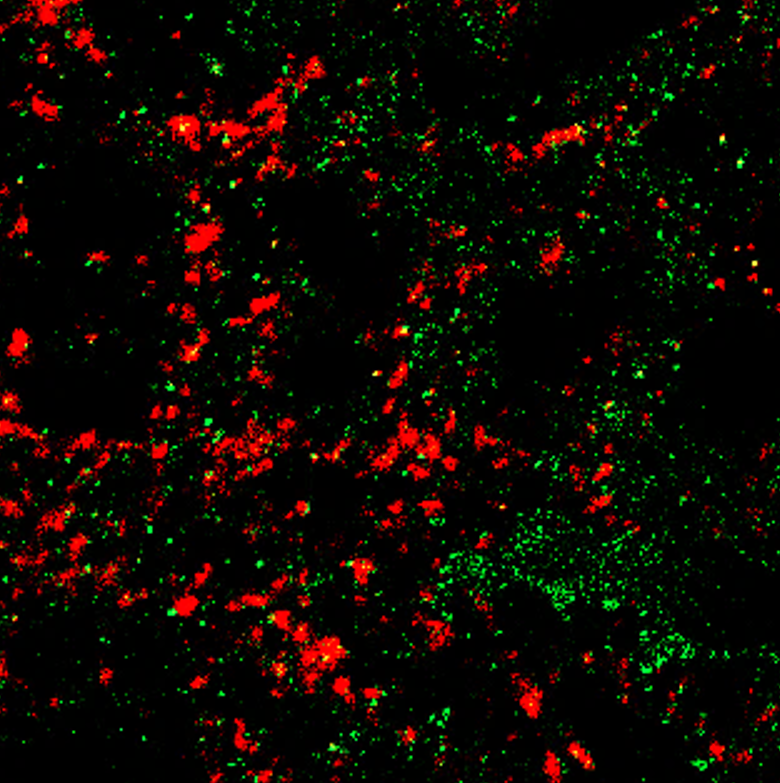

Supplement: Supplementary file 6 — Source data Fig. 3 [file 44319_2025_452_MOESM6_ESM.zip › Figure 3/Figure 3J/St3gal2,3 dKO_Sham_ipsi.tif]

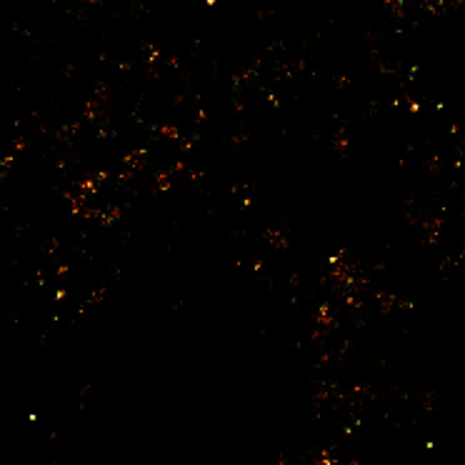

Supplement: Supplementary file 6 — Source data Fig. 3 [file 44319_2025_452_MOESM6_ESM.zip › Figure 3/Figure 3K/St3gal2,3 dKO_3d merged.tif]

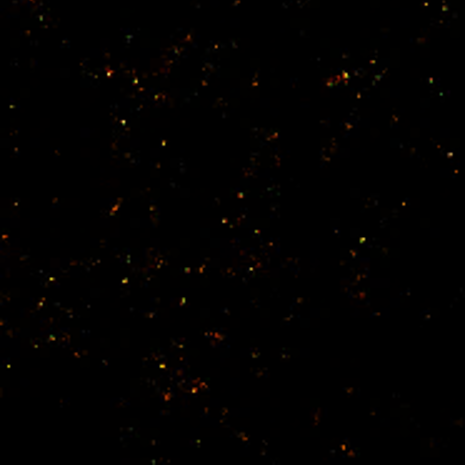

Supplement: Supplementary file 6 — Source data Fig. 3 [file 44319_2025_452_MOESM6_ESM.zip › Figure 3/Figure 3K/St3gal2,3 dKO_Sham merged.tif]

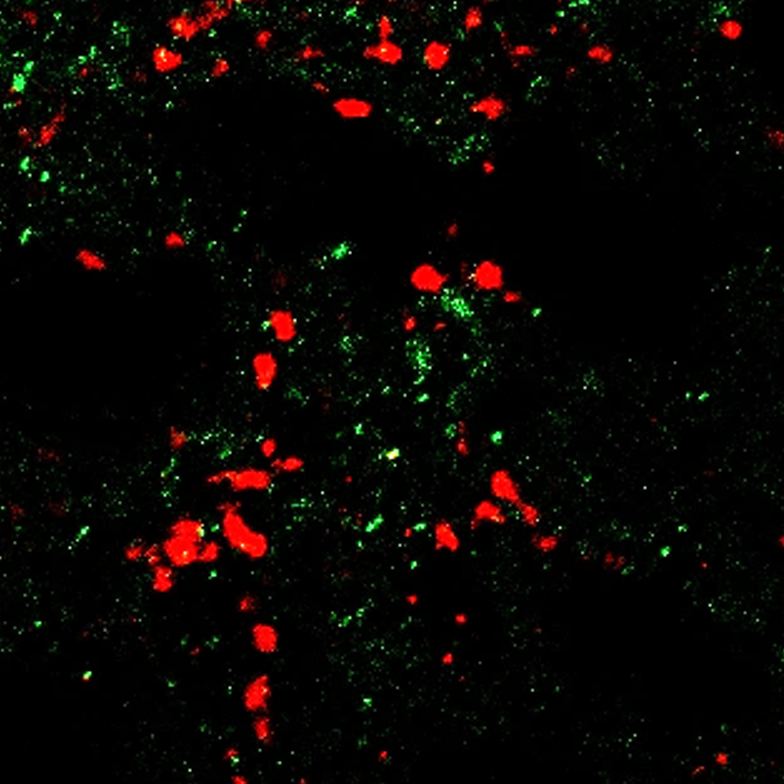

Supplement: Supplementary file 6 — Source data Fig. 3 [file 44319_2025_452_MOESM6_ESM.zip › Figure 3/Figure 3M/St3gal2 cKO_cont.tif]

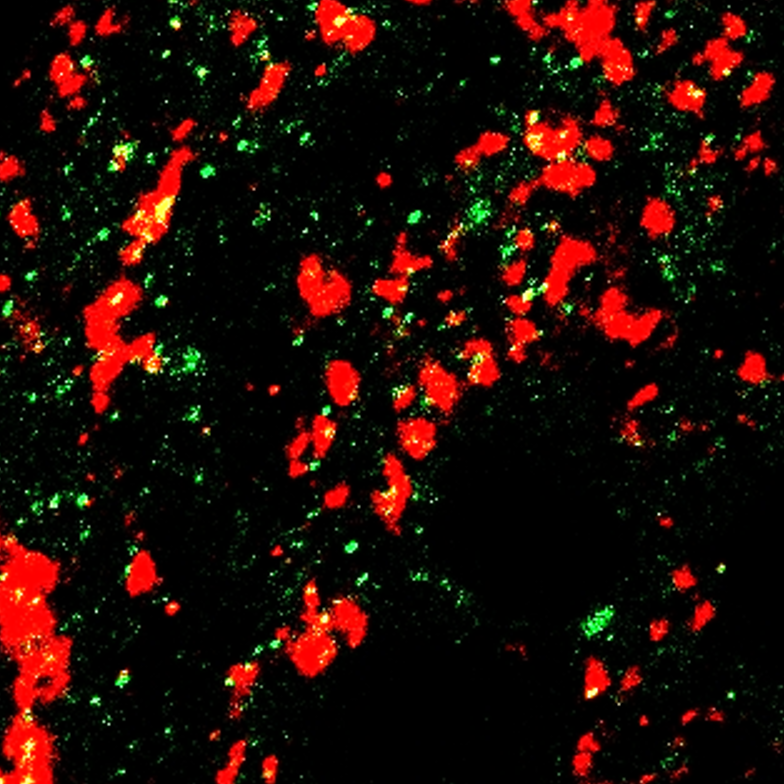

Supplement: Supplementary file 6 — Source data Fig. 3 [file 44319_2025_452_MOESM6_ESM.zip › Figure 3/Figure 3M/St3gal2 cKO_ipsi.tif]

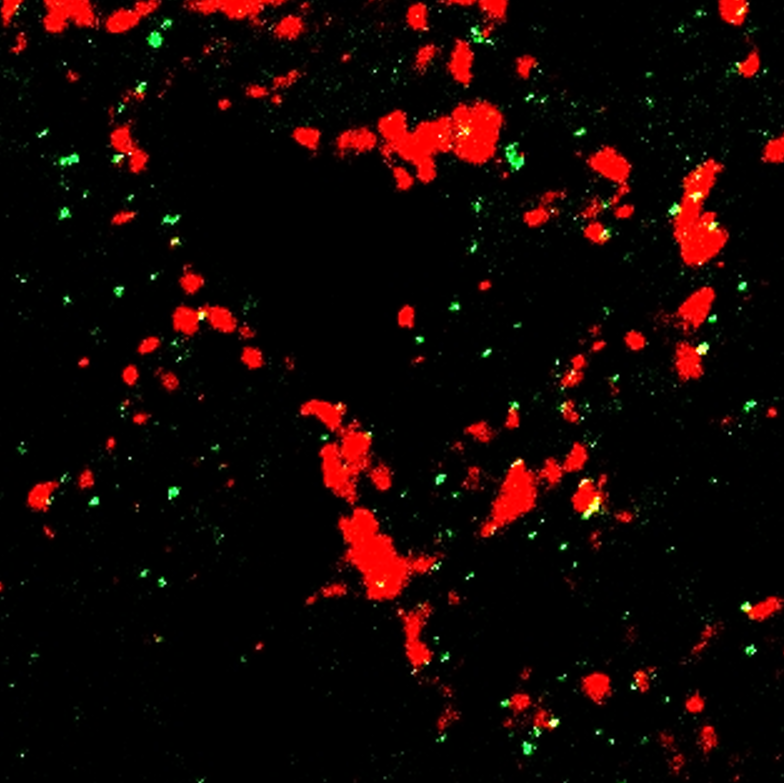

Supplement: Supplementary file 6 — Source data Fig. 3 [file 44319_2025_452_MOESM6_ESM.zip › Figure 3/Figure 3M/St3gal2 floxed_cont.tif]

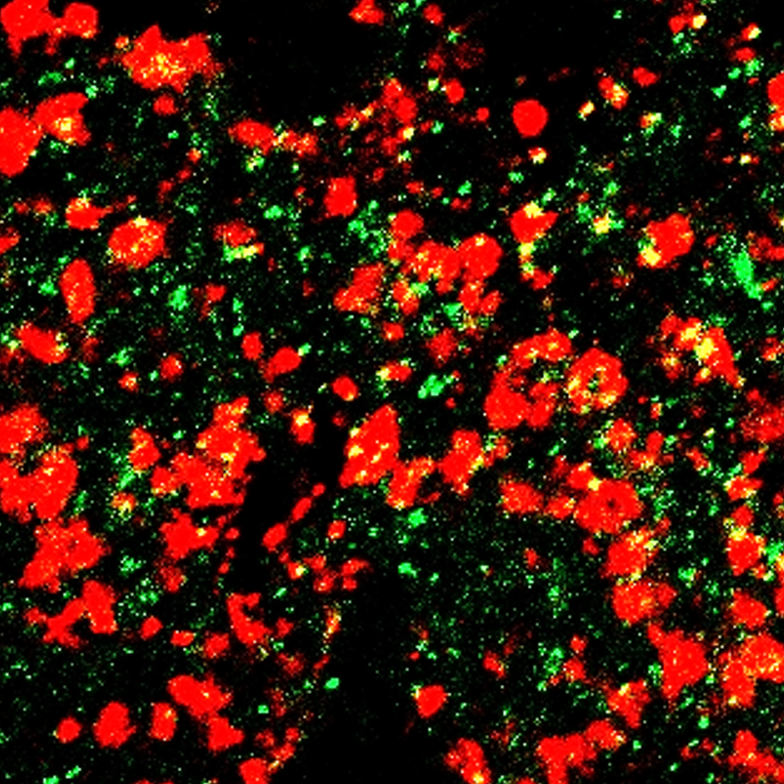

Supplement: Supplementary file 6 — Source data Fig. 3 [file 44319_2025_452_MOESM6_ESM.zip › Figure 3/Figure 3M/St3gal2 floxed_ipsi.tif]

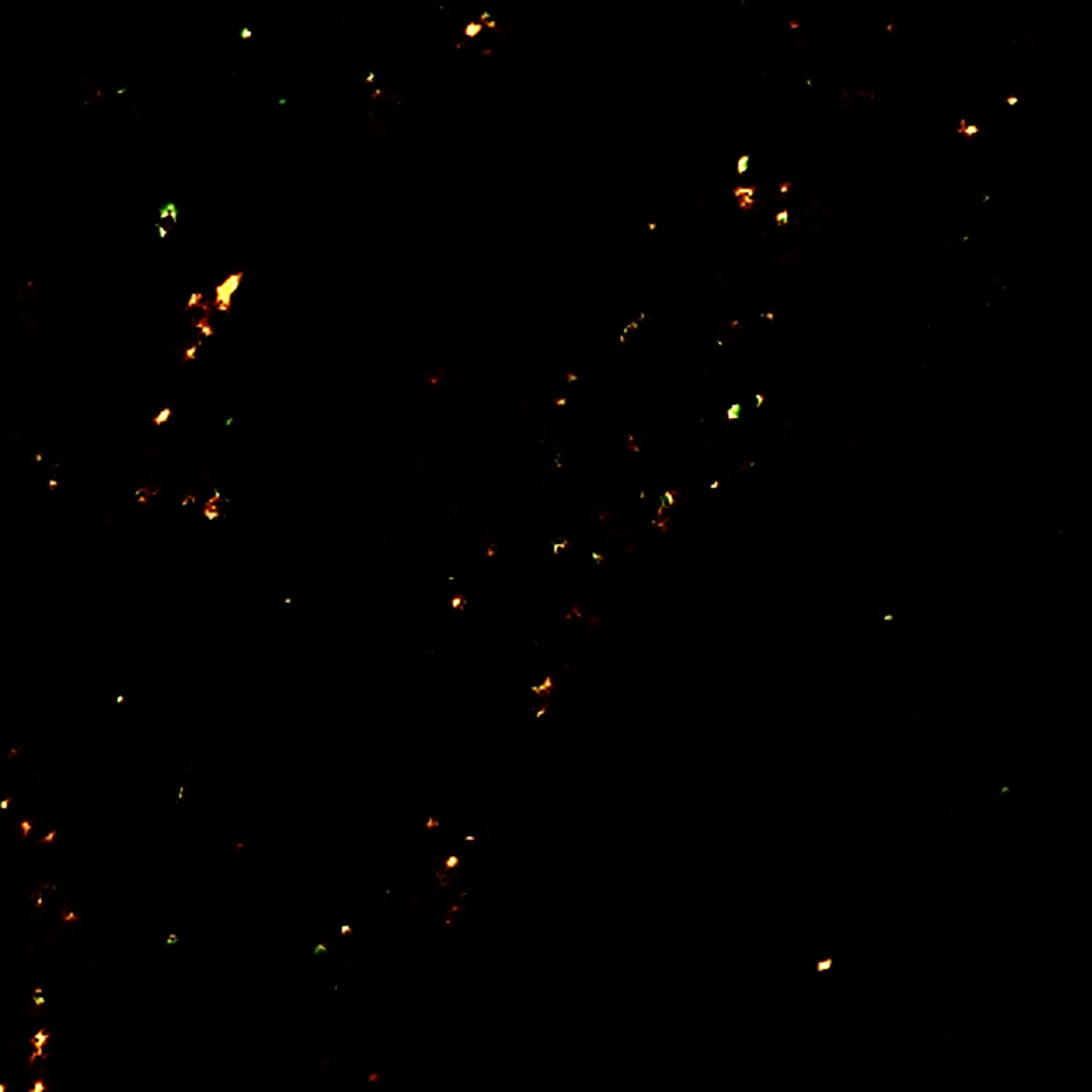

Supplement: Supplementary file 6 — Source data Fig. 3 [file 44319_2025_452_MOESM6_ESM.zip › Figure 3/Figure 3N/st3gal2 cKO merged.tif]

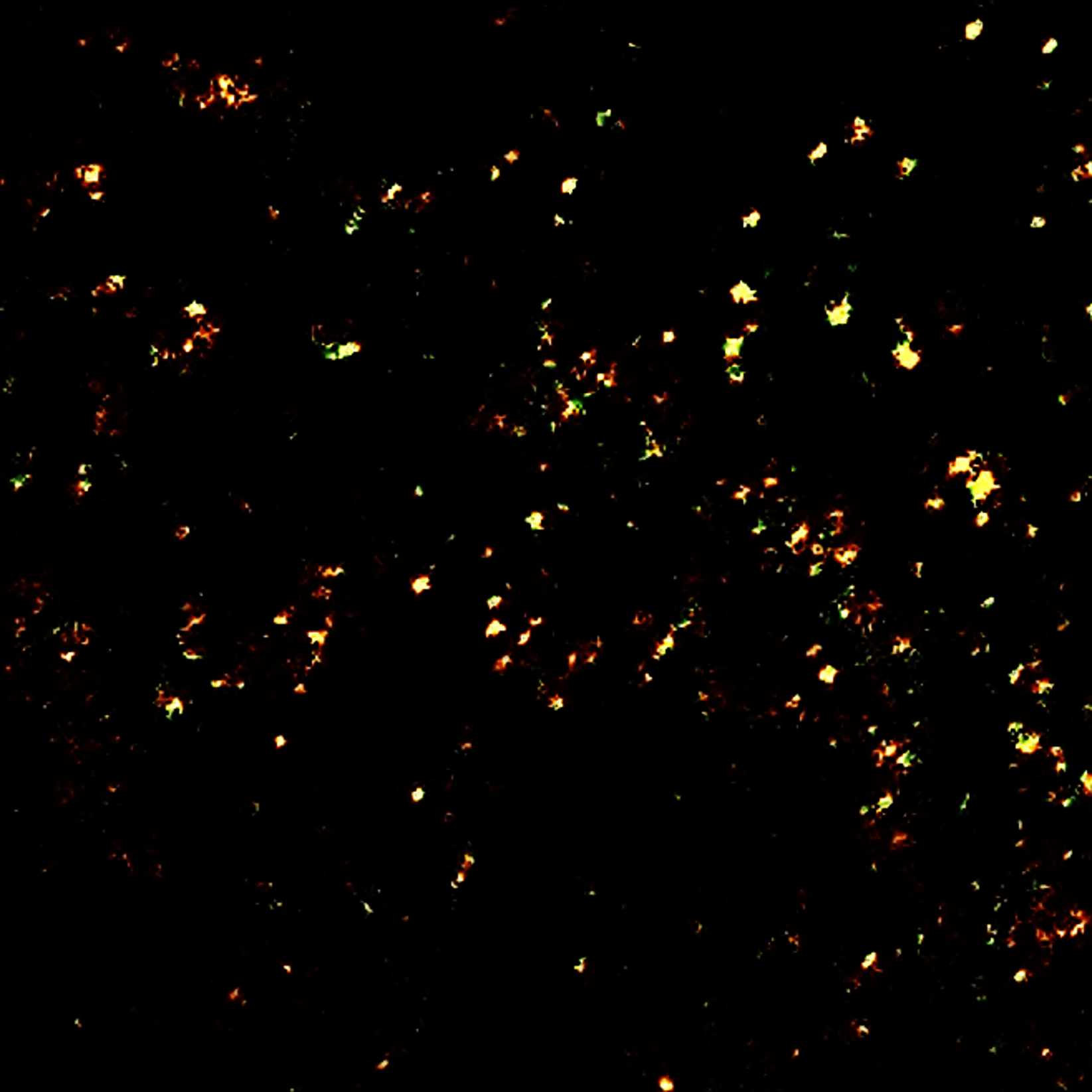

Supplement: Supplementary file 6 — Source data Fig. 3 [file 44319_2025_452_MOESM6_ESM.zip › Figure 3/Figure 3N/st3gal2 floxed merged.tif]

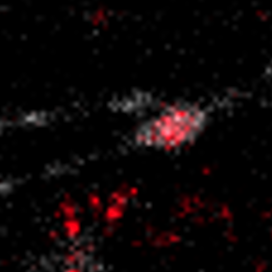

Supplement: Supplementary file 7 — Source data Fig. 4 [file 44319_2025_452_MOESM7_ESM.zip › Figure 4/Figure 4C/GT1b+_1.tif]

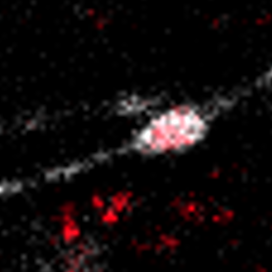

Supplement: Supplementary file 7 — Source data Fig. 4 [file 44319_2025_452_MOESM7_ESM.zip › Figure 4/Figure 4C/GT1b+_2.tif]

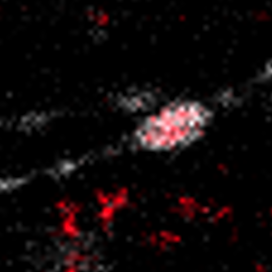

Supplement: Supplementary file 7 — Source data Fig. 4 [file 44319_2025_452_MOESM7_ESM.zip › Figure 4/Figure 4C/GT1b+_3.tif]

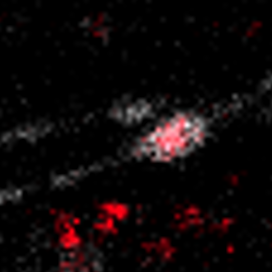

Supplement: Supplementary file 7 — Source data Fig. 4 [file 44319_2025_452_MOESM7_ESM.zip › Figure 4/Figure 4C/GT1b+_4.tif]

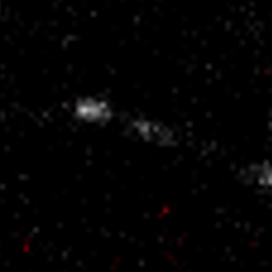

Supplement: Supplementary file 7 — Source data Fig. 4 [file 44319_2025_452_MOESM7_ESM.zip › Figure 4/Figure 4C/GT1b-_1.tif]

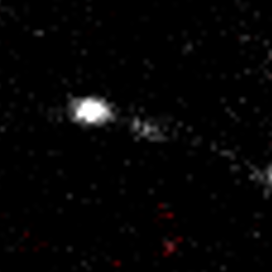

Supplement: Supplementary file 7 — Source data Fig. 4 [file 44319_2025_452_MOESM7_ESM.zip › Figure 4/Figure 4C/GT1b-_2.tif]

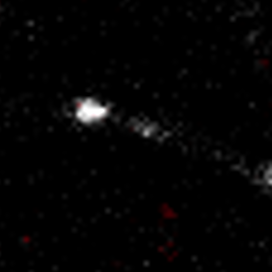

Supplement: Supplementary file 7 — Source data Fig. 4 [file 44319_2025_452_MOESM7_ESM.zip › Figure 4/Figure 4C/GT1b-_3.tif]

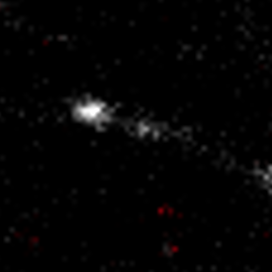

Supplement: Supplementary file 7 — Source data Fig. 4 [file 44319_2025_452_MOESM7_ESM.zip › Figure 4/Figure 4C/GT1b-_4.tif]

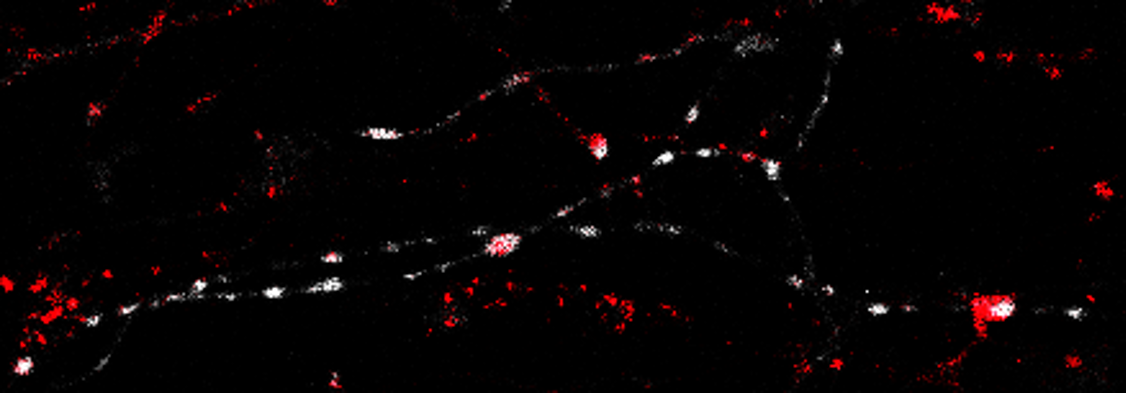

Supplement: Supplementary file 7 — Source data Fig. 4 [file 44319_2025_452_MOESM7_ESM.zip › Figure 4/Figure 4C/Merged image.tif]

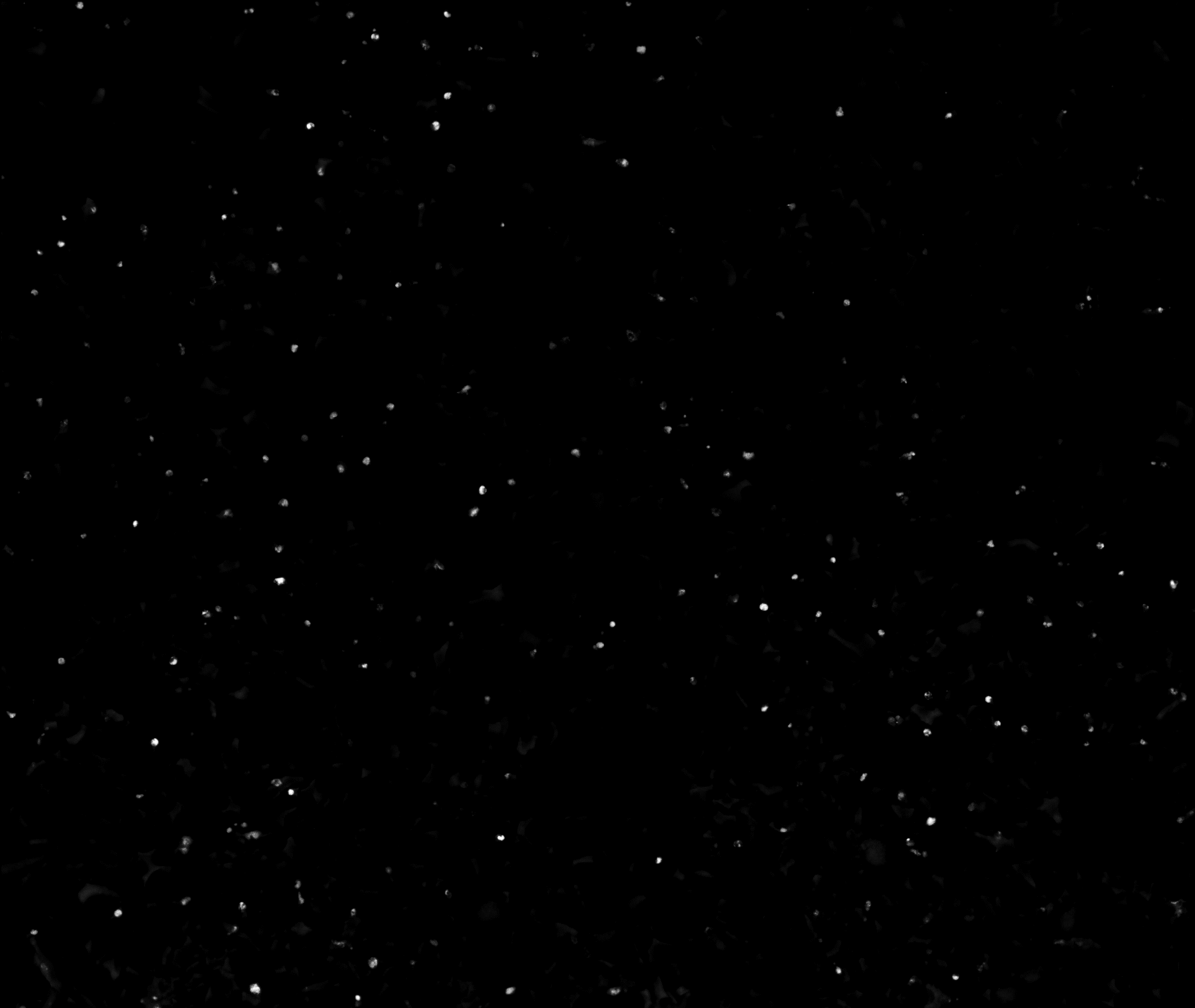

Supplement: Supplementary file 8 — Source data Fig. 5 [file 44319_2025_452_MOESM8_ESM.zip › Figure 5/Figure 5C/Cont_10'.tif]

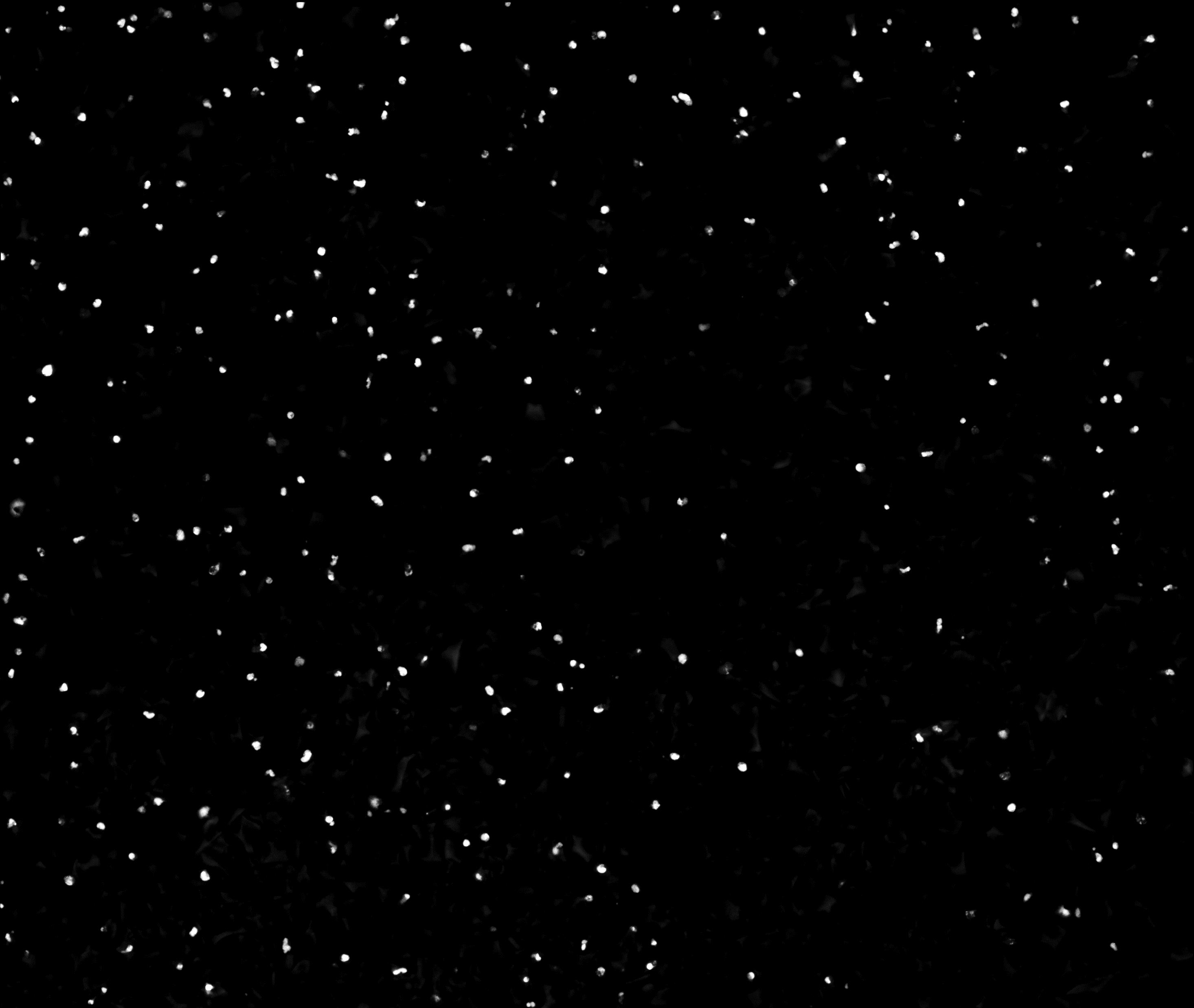

Supplement: Supplementary file 8 — Source data Fig. 5 [file 44319_2025_452_MOESM8_ESM.zip › Figure 5/Figure 5C/Cont_30'.tif]

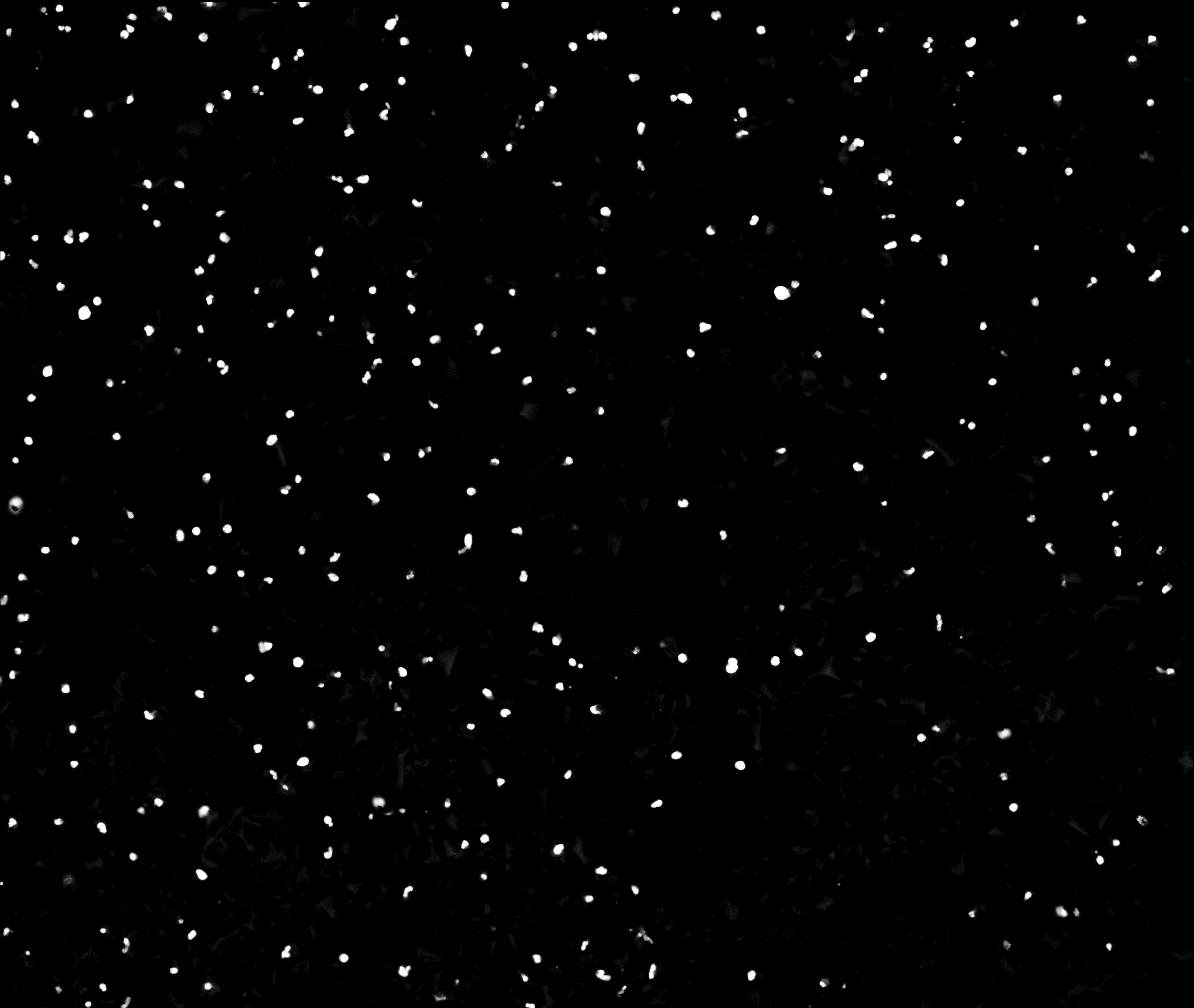

Supplement: Supplementary file 8 — Source data Fig. 5 [file 44319_2025_452_MOESM8_ESM.zip › Figure 5/Figure 5C/Cont_60'.tif]

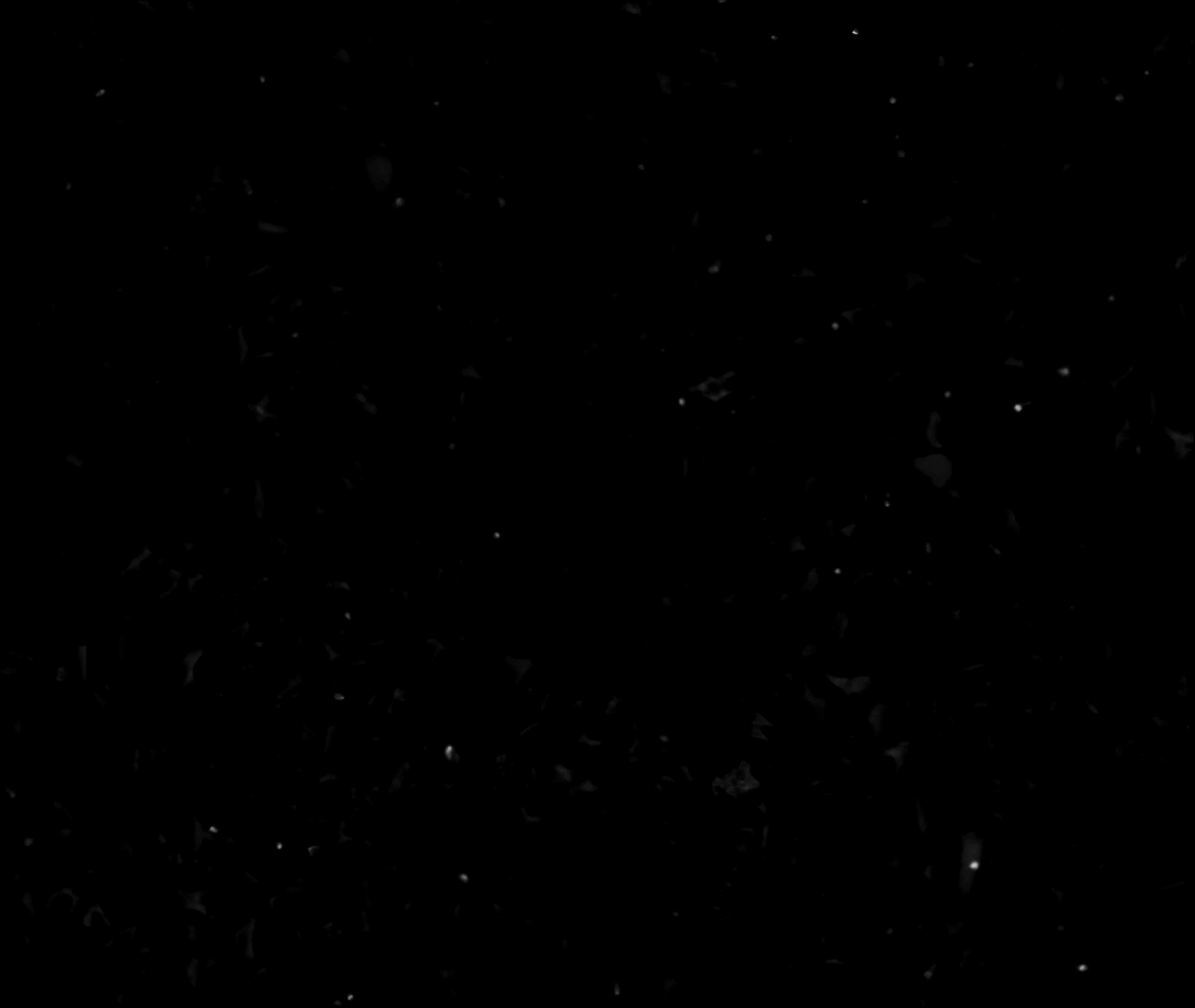

Supplement: Supplementary file 8 — Source data Fig. 5 [file 44319_2025_452_MOESM8_ESM.zip › Figure 5/Figure 5C/GT1b 100_10'.tif]
