# Supplementary figures and images for: Ganglioside GT1b prevents selective spinal synapse removal following peripheral nerve injury (part 2 of 2)
Source: EMBO Rep. 2025 Apr 30;26(12):2994–3023. doi: 10.1038/s44319-025-00452-2 (PMC12187942; doi:10.1038/s44319-025-00452-2)

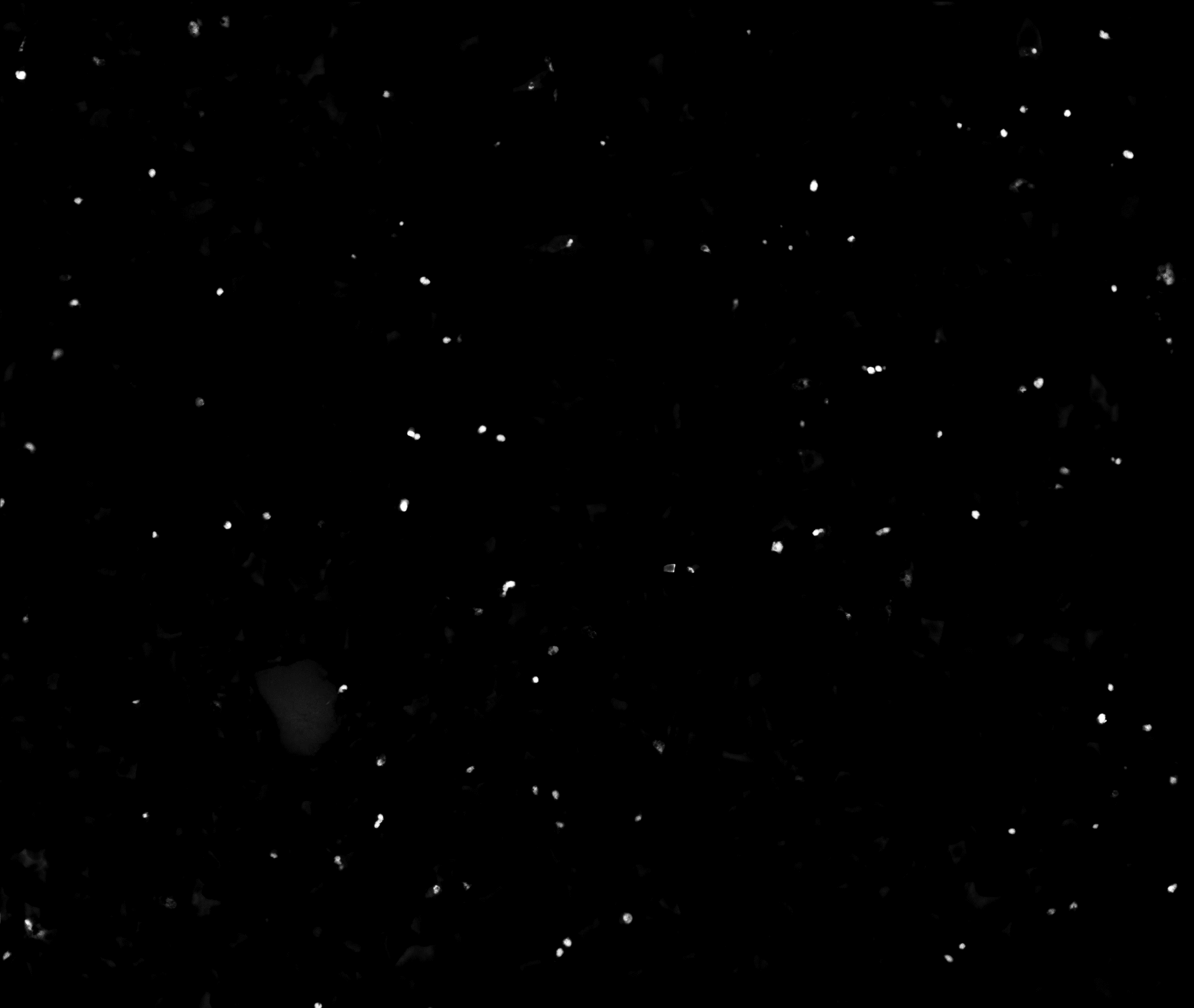

Supplement: Supplementary file 8 — Source data Fig. 5 [file 44319_2025_452_MOESM8_ESM.zip › Figure 5/Figure 5C/GT1b 100_30'.tif]

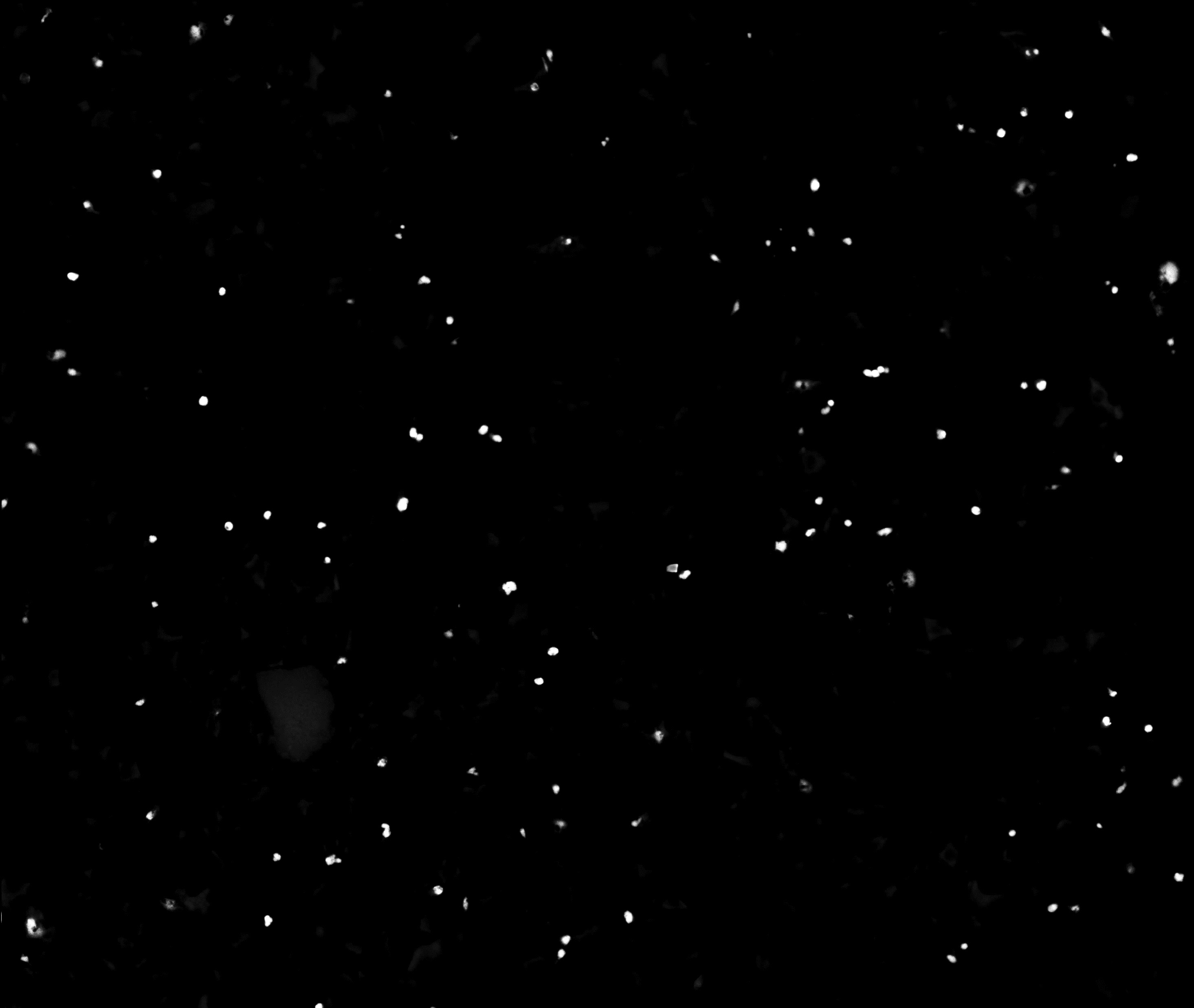

Supplement: Supplementary file 8 — Source data Fig. 5 [file 44319_2025_452_MOESM8_ESM.zip › Figure 5/Figure 5C/GT1b 100_60'.tif]

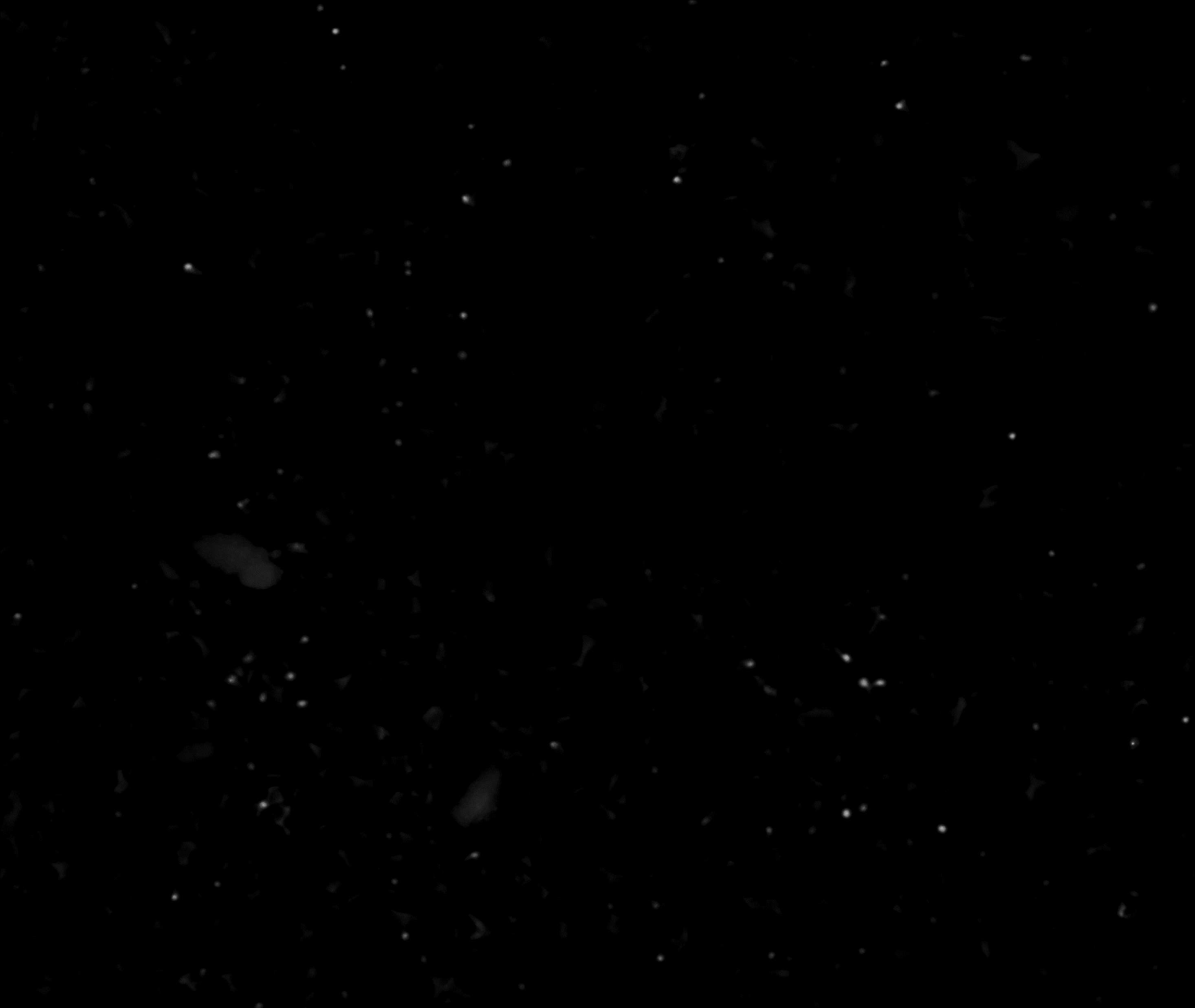

Supplement: Supplementary file 8 — Source data Fig. 5 [file 44319_2025_452_MOESM8_ESM.zip › Figure 5/Figure 5C/GT1b 10_10'.tif]

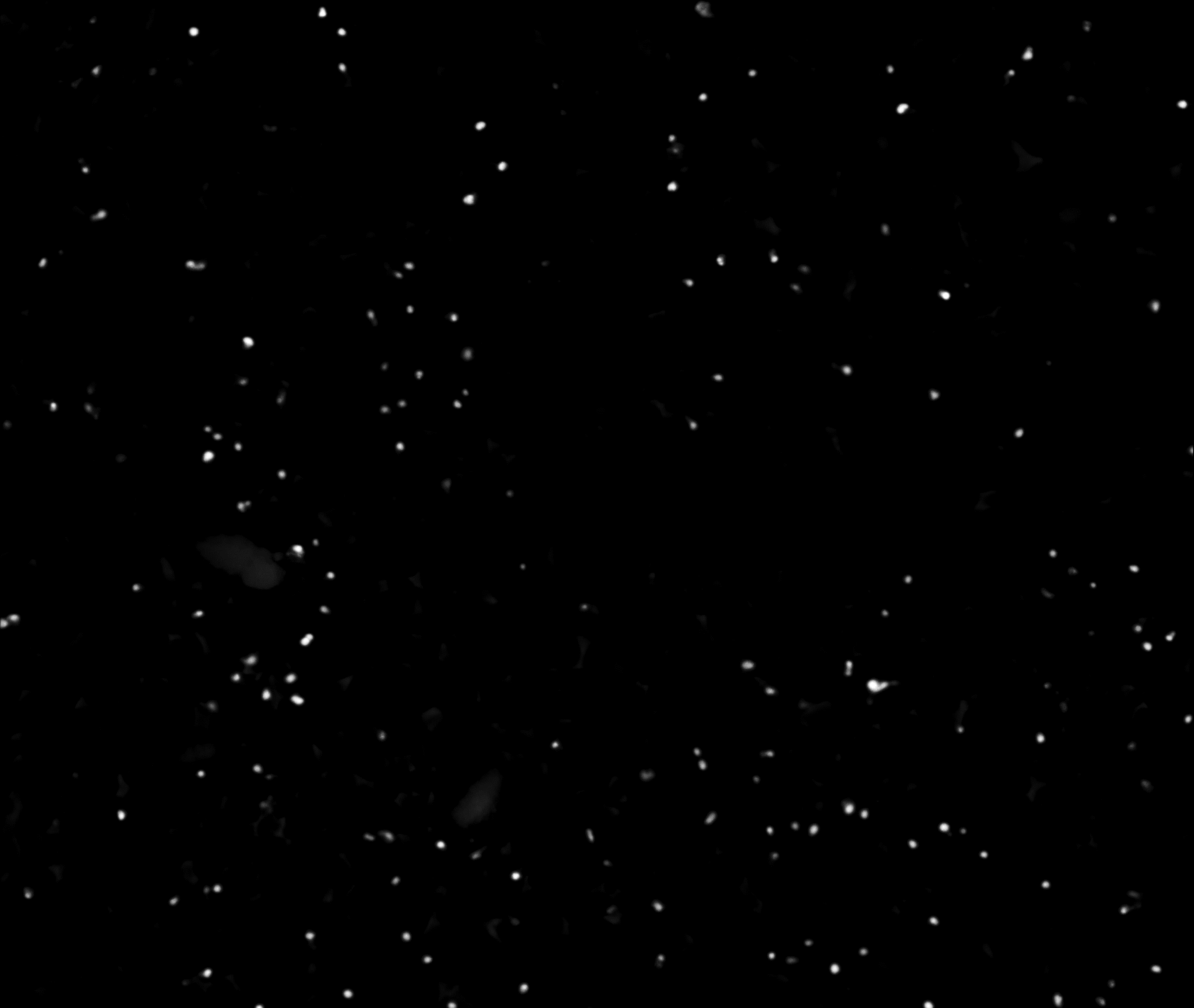

Supplement: Supplementary file 8 — Source data Fig. 5 [file 44319_2025_452_MOESM8_ESM.zip › Figure 5/Figure 5C/GT1b 10_30'.tif]

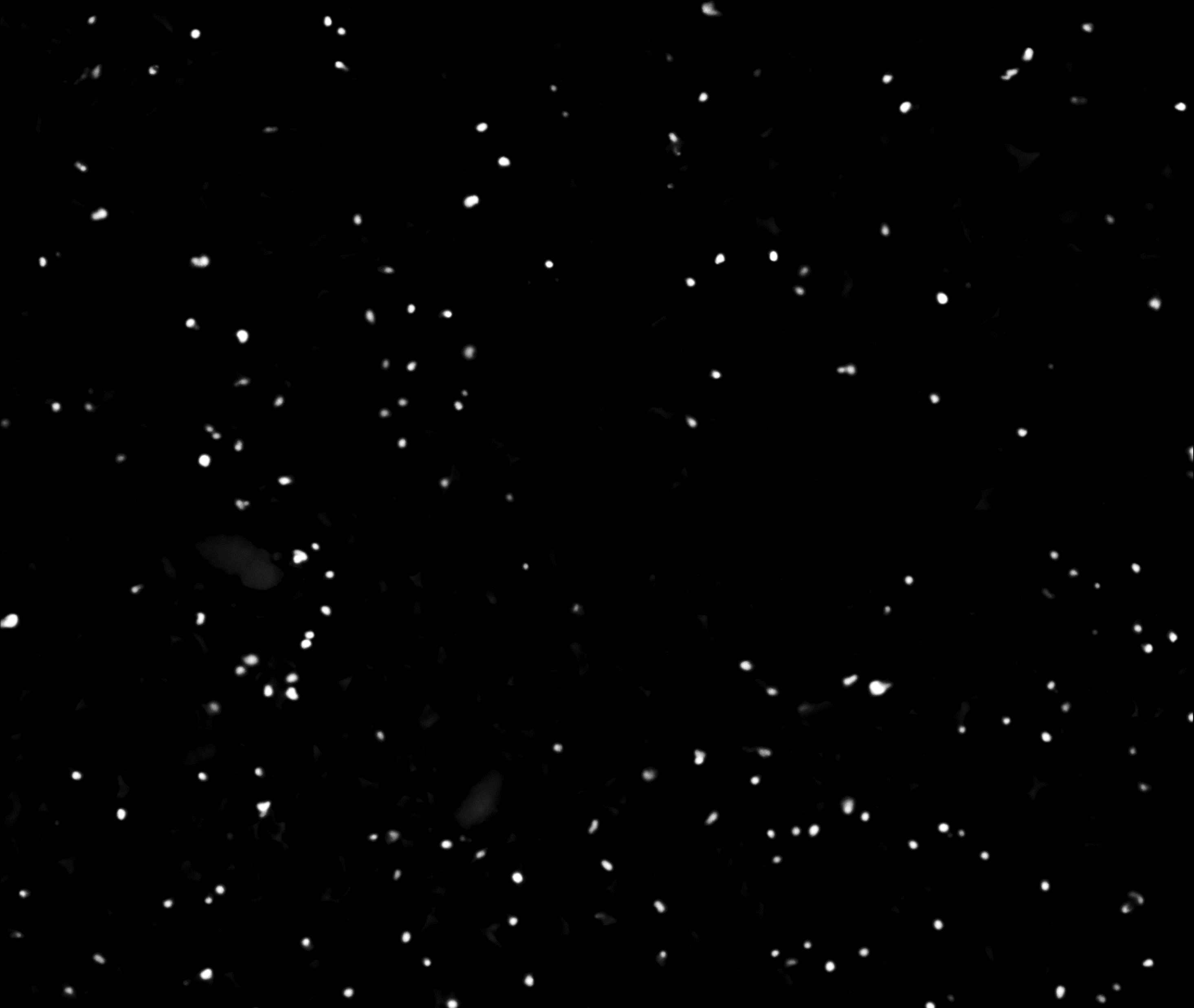

Supplement: Supplementary file 8 — Source data Fig. 5 [file 44319_2025_452_MOESM8_ESM.zip › Figure 5/Figure 5C/GT1b 10_60'.tif]

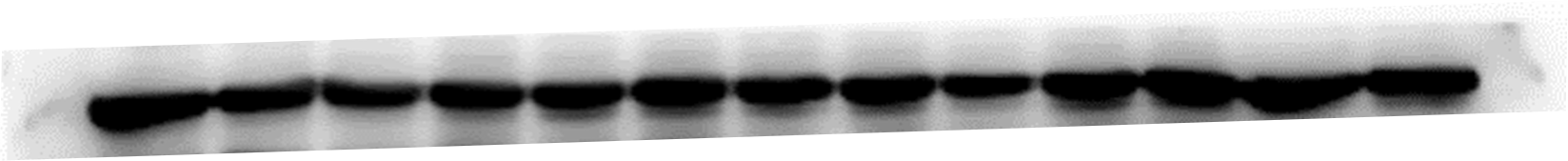

Supplement: Supplementary file 8 — Source data Fig. 5 [file 44319_2025_452_MOESM8_ESM.zip › Figure 5/Figure 5I/GAPDH.tif]

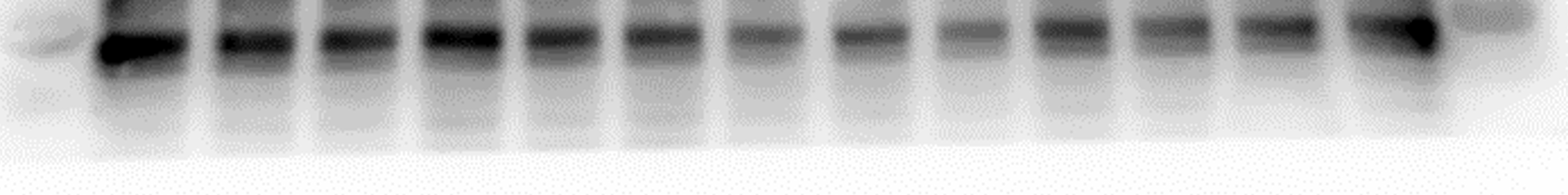

Supplement: Supplementary file 8 — Source data Fig. 5 [file 44319_2025_452_MOESM8_ESM.zip › Figure 5/Figure 5I/p-SYK.tif]

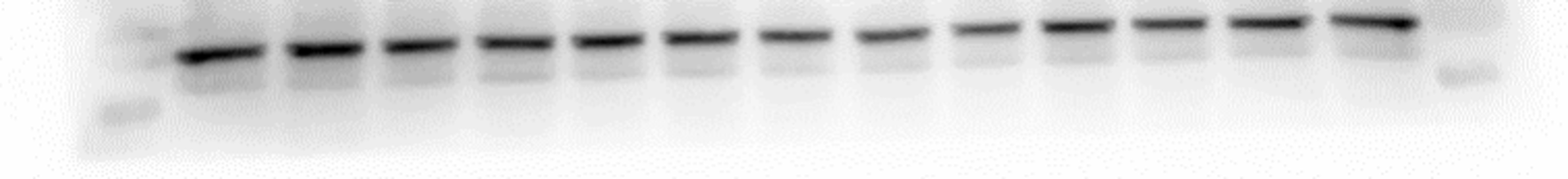

Supplement: Supplementary file 8 — Source data Fig. 5 [file 44319_2025_452_MOESM8_ESM.zip › Figure 5/Figure 5I/SYK.tif]
