# Supplementary material for: Ganglioside GT1b prevents selective spinal synapse removal following peripheral nerve injury
Source: EMBO Rep. 2025 Apr 30;26(12):2994–3023. doi: 10.1038/s44319-025-00452-2 (PMC12187942; doi:10.1038/s44319-025-00452-2)
Supplement: Supplementary file 9 — Expanded View Figures [file 44319_2025_452_MOESM9_ESM.pdf]

# Expanded View Figures

**Figure EV1. AAV-based pH-reporter expression in each type of synapse and its attachment to spinal glia.**

(**A**) Targeted expression of the synapse type–specific pH-reporter system, which was introduced via AAV vectors. Representative immunostained images demonstrate the colocalization of the pH-reporter with each synaptic marker. Scale bar = 10 μm. (**B**) Comparative analysis of ExPre attachment to each glial cell type. *n*; Sham = 8, 3 d = 15, 7 d = 7. (**C**) Comparative analysis of ExPost attachment to each glial cell type. *P* value (Sham); Microglia vs. Astrocyte <0.0001. *P* value (3 d); Microglia vs. Astrocyte <0.0001. *n*; Sham = 9, 3 d = 9, 7 d = 6. (**D**) Comparative analysis of InhiPre attachment to each glial cell type. *P* value (Sham); Microglia vs. Astrocyte = 0.0272. *n*; Sham = 6, 3 d = 7, 7 d = 6. (**E**) Comparative analysis of InhiPost attachment to each glial cell type. *P* value (Sham); Microglia vs. Astrocyte = 0.0004. *P* value (3 d); Microglia vs. Astrocyte = 0.0418. P value (7 d); Microglia vs. Astrocyte <0.0001. *n*; Sham = 8, 3 d = 7, 7 d = 8. Data are represented as the mean ± SEM; *$P < 0.05$, ***$P < 0.001$, ****$P < 0.0001$; two-way ANOVA with Tukey's multiple comparison test.

© The Author(s)

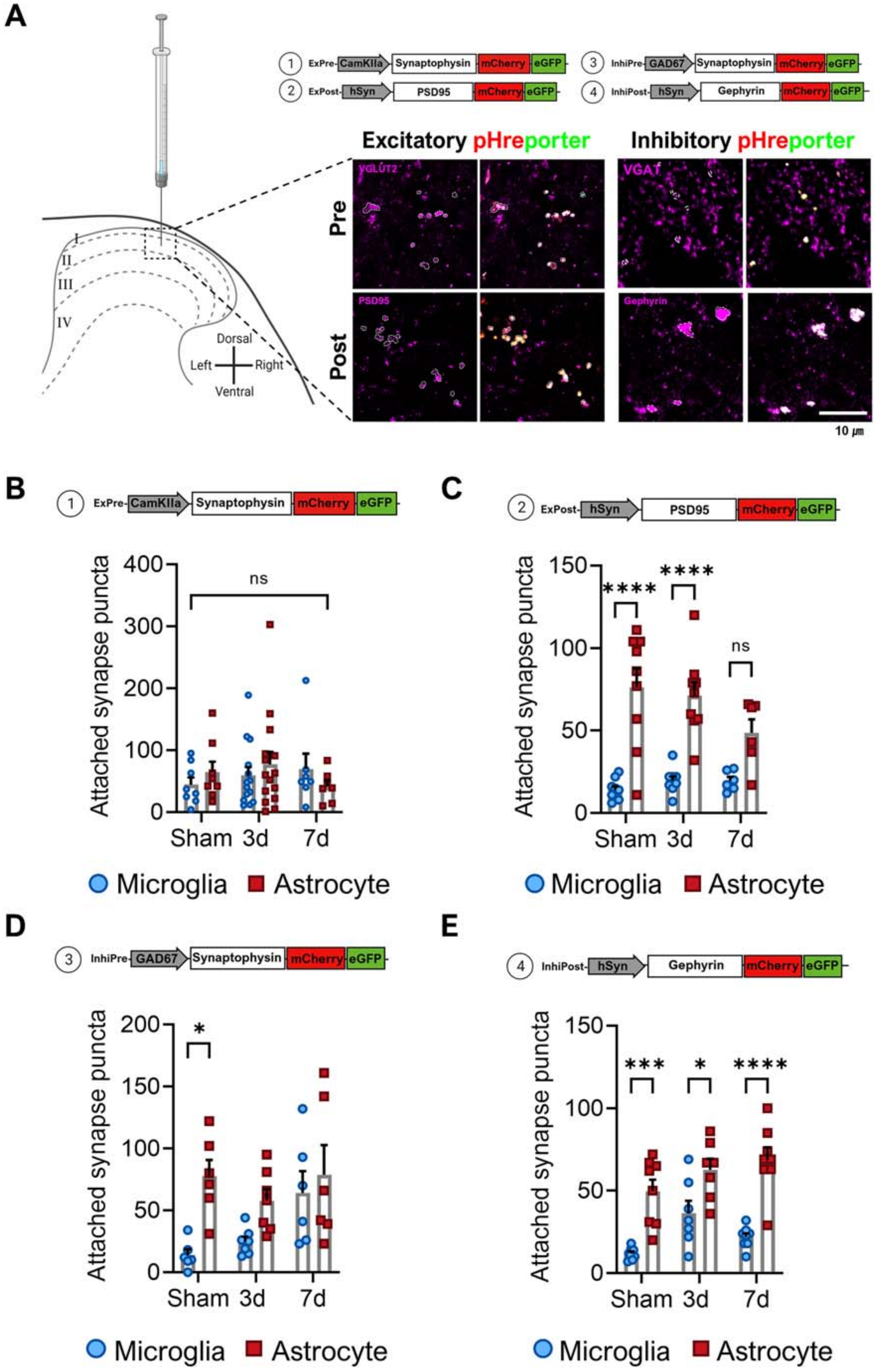
A
1 ExPre- CamKIIa Synaptophysin mCherry eGFP
2 ExPost- hSyn PSD95 mCherry eGFP
3 InhiPre- GAD67 Synaptophysin mCherry eGFP
4 InhiPost- hSyn Gephyrin mCherry eGFP
Excitatory pHreporter
Inhibitory pHreporter
VGLUT2
VGAT
PSD95
Gephyrin
Pre
Post
I
II
III
IV
Dorsal
Left
Right
Ventral
10 µm
B
C
D
E
Attached synapse puncta
ns
****
*
***
Sham
3d
7d
Microglia
Astrocyte

 © The Author(s)

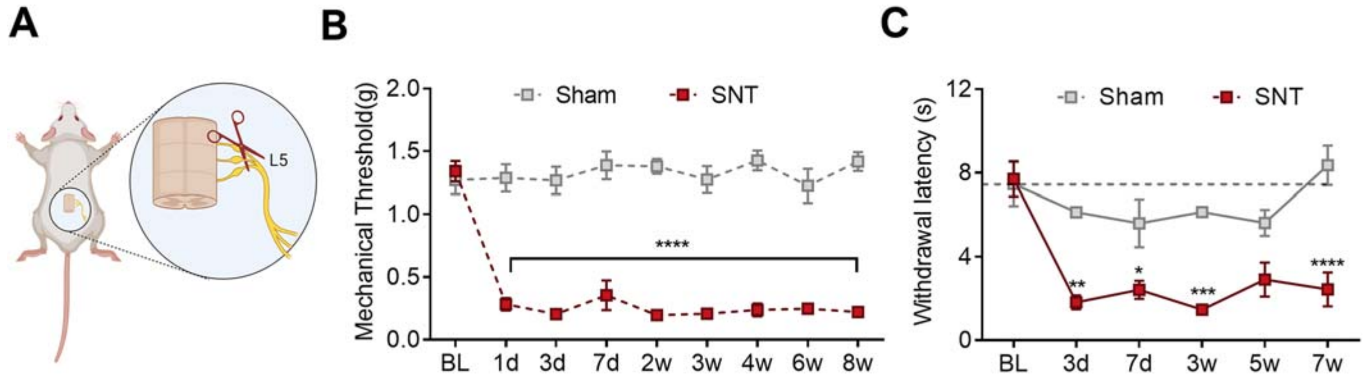


**Figure EV2. L5 spinal nerve transection (L5 SNT) model of neuropathic pain.**

(**A**) Schematic of the L5 SNT model used to induce neuropathic pain in mice. (**B**) Assessment of mechanical allodynia and (**C**) thermal hyperalgesia. *P* value (mechanical allodynia); Sham vs. SNT < 0.0001. *n* (mechanical allodynia); Sham = 9, SNT = 16. *P* value (thermal hyperalgesia); 3 d:Sham vs. 3 d:SNT = 0.0016, 7 d:Sham vs. 7 d:SNT = 0.0256, 3w:Sham vs. 3w:SNT = 0.0007, 5w:Sham vs. 5w:SNT = 0.0779, 7w:Sham vs. 7w:SNT < 0.0001. Sham = 3, SNT = 3. Data are represented as the mean ± SEM; *$P < 0.05$, ***$P < 0.001$, ****$P < 0.0001$; two-way ANOVA with Bonferroni's multiple comparison test.

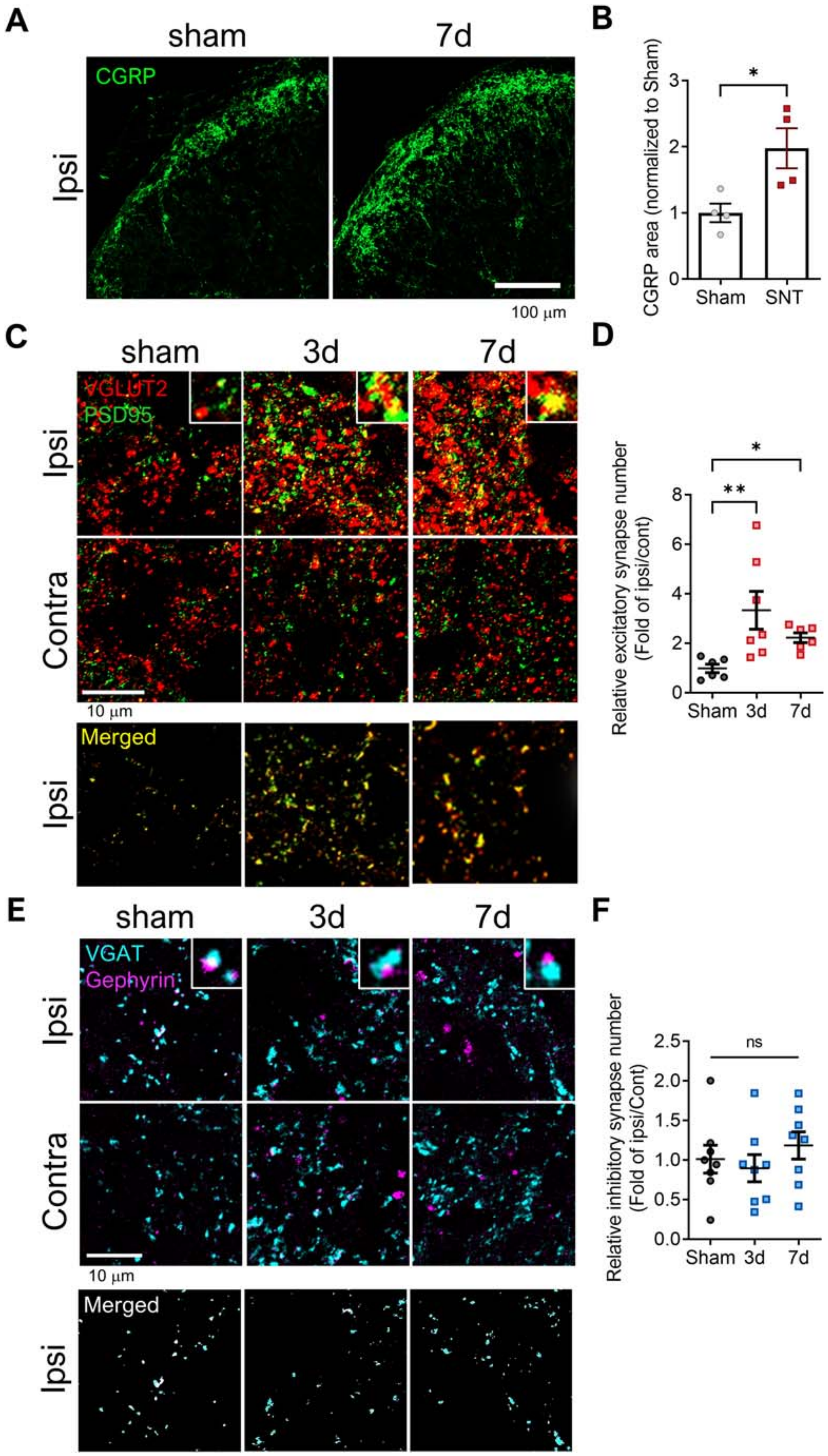
A
sham
7d
CGRP
Ipsi
100 μm
B
CGRP area (normalized to Sham)
*
Sham
SNT
C
sham
3d
7d
VGLUT2
PSD95
Ipsi
Contra
10 μm
Merged
Ipsi
D
Relative excitatory synapse number (Fold of ipsi/cont)
**
*
Sham
3d
7d
E
sham
3d
7d
VGAT
Gephyrin
Ipsi
Contra
10 μm
Merged
Ipsi
F
Relative inhibitory synapse number (Fold of ipsi/Cont)
ns
Sham
3d
7d

 © The Author(s)

**Figure EV3. Expansion of excitatory synapses in the superficial layer (LI–II) of the spinal cord.**

(**A**, **B**) Immunofluorescence visualization of calcitonin gene-related peptide (CGRP) in the dorsal horn, depicting and quantifying the expansion of afferent terminals after nerve injury. Scale bar = 100 µm. *P* value; Sham vs. SNT = 0.0267. *n*; Sham = 4, SNT = 4. (**C**) High-resolution confocal images of spinal cord sections stained for VGLUT2 (red) and PSD95 (green) to label excitatory synapses. Insets show a magnified view of co-localized VGLUT2 + PSD95+ puncta. (**D**) Quantification of co-localized excitatory synaptic puncta following SNT. *P* value; Sham vs. 3 d = 0.0036, Sham vs. 7 d = 0.0153. *n*; Sham = 6, 3 d = 7, 7 d = 6. Scale bar = 10 µm. (**E**) High-resolution confocal images of spinal cord sections stained for VGAT (cyan) and gephyrin (magenta) to label inhibitory synapses. Insets show magnified view of co-localized VGAT+ gephyrin+ puncta. (**F**) Quantification of co-localized inhibitory synaptic puncta after SNT. Scale bar = 10 µm. *n*; Sham = 8, 3 d = 8, 7 d = 8. Data are presented as the mean ± SEM; *$P < 0.05$, **$P < 0.01$; one-way ANOVA with Dunnett's multiple comparison test and Student's *t* test.

© The Author(s)

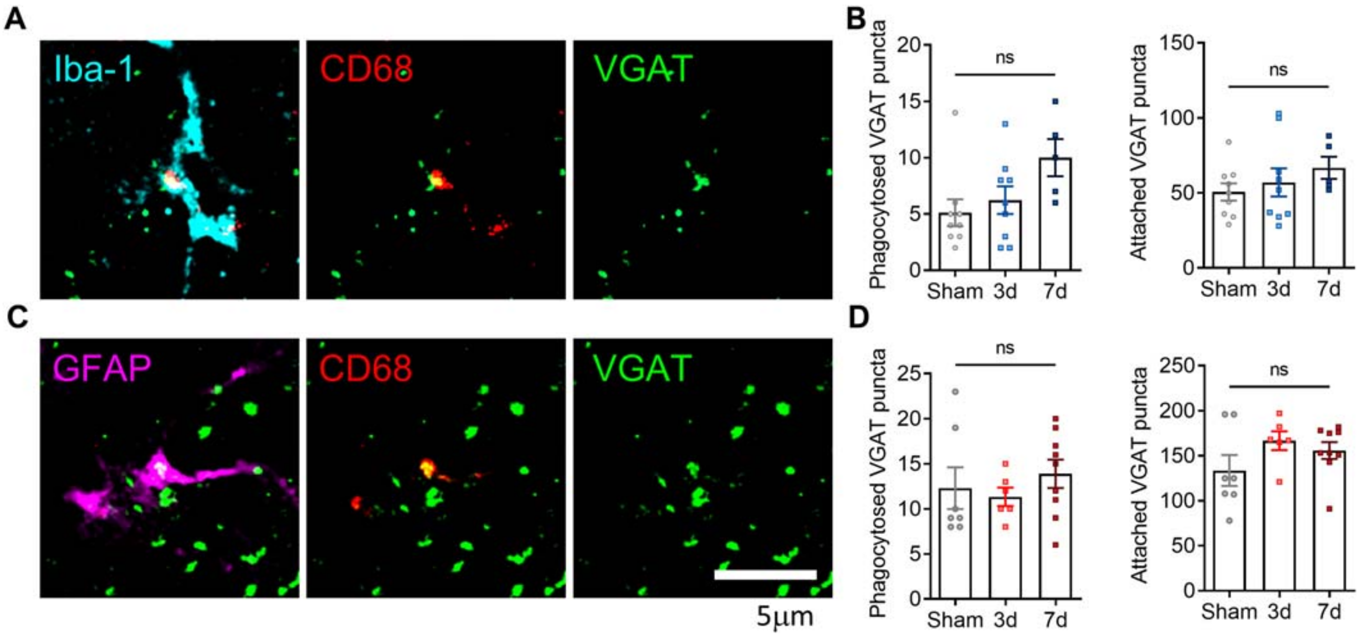


**Figure EV4. Glial phagocytosis of VGAT-positive InhiPre after peripheral nerve injury.**

(**A**) Representative immunostained spinal cord section showing VGAT, CD68 and Iba-1, highlighting microglial phagocytosis of VGAT-positive InhiPre. (**B**) Microglial phagocytosis and surface attachment of VGAT-positive InhiPre. *n*; Sham = 9, 3 d = 9, 7 d = 5. (**C**) Representative immunostained spinal cord section showing VGAT, CD68 and GFAP, illustrating astrocytic phagocytosis of VGAT-positive InhiPre. (**D**) Astrocytic phagocytosis and surface attachment of VGAT-positive InhiPre. *n*; Sham = 7, 3 d = 6, 7 d = 9. Scale bar = 5 μm. Data are represented as the mean ± SEM; one-way ANOVA with Dunnett's multiple comparison test.

 © The Author(s)

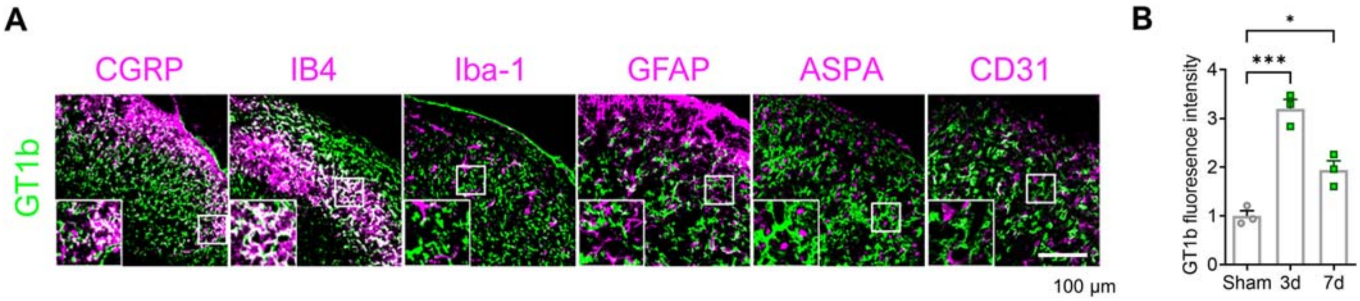


**Figure EV5. Localization of GT1b in spinal afferent terminals and its accumulation following peripheral nerve injury.**

(**A**) Immunostained spinal cord sections demonstrating GT1b localization with markers for various cell types: CGRP (calcitonin gene-related peptide) for peptidergic afferents, IB4 (isolectin B4) for non-peptidergic afferents, Iba-1 for microglia, GFAP for astrocytes, ASAP (aspartoacylase) for oligodendrocytes, and CD31 for endothelia. (**B**) GT1b fluorescence intensity across post-injury time points in the dorsal horn of the spinal cord. *P* value; Sham vs. 3 d = 0.0002, Sham vs. 7 d = 0.0128. *n*; Sham = 3, 3 d = 3, 7 d = 3. Scale bar = 100 μm. Data are represented as the mean ± SEM; *$P < 0.05$, ***$P < 0.001$; one-way ANOVA with Dunnett's multiple comparison test.

© The Author(s)

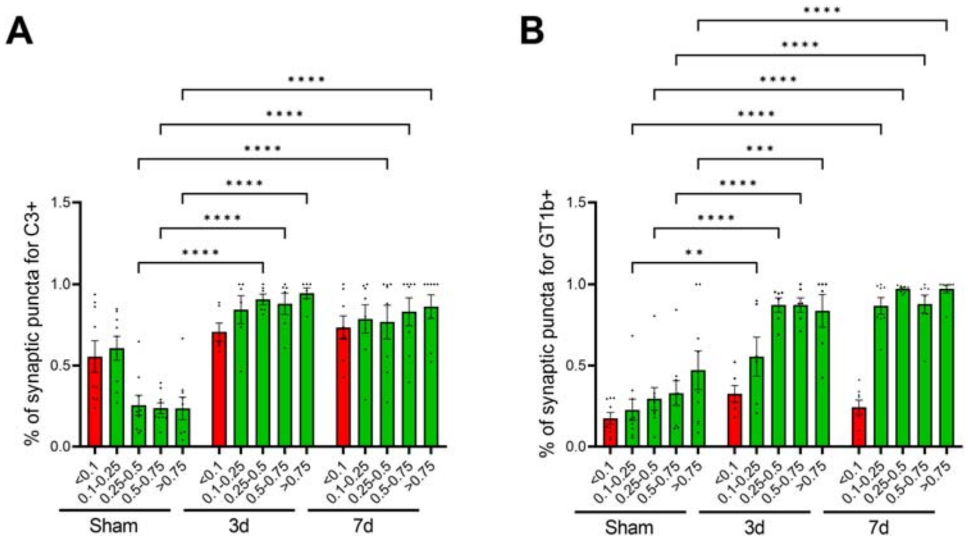


**Figure EV6. EGFP intensity-dependent ratios of C3- and GT1b-positive synaptic puncta.**

Five categories of ExPre synaptic puncta were defined based on the intensity of EGFP. mCherry puncta with less than 10% of EGFP were classified as phagocytosed puncta, and the remaining puncta were divided into four groups (10–25%, 25–50%, 50–75%, and more than 75% EGFP co-localized with mCherry puncta). (**A**) Relative ratio of C3-positive puncta in each synapse category. *P* value (0.25-0.5); Sham vs. 3 d < 0.0001, Sham vs. 7 d < 0.0001. *P* value (0.5-0.75); Sham vs. 3 d < 0.0001, Sham vs. 7 d < 0.0001. *P* value (>0.75); Sham vs. 3 d < 0.0001, Sham vs. 7 d < 0.0001. (**B**) Relative ratio of GT1b-positive puncta in each synapse category. *P* value (0.1-0.25); Sham vs. 3 d = 0.0029, Sham vs. 7 d < 0.0001. *P* value (0.25-0.5); Sham vs. 3 d < 0.0001, Sham vs. 7 d < 0.0001. *P* value (0.5-0.75); Sham vs. 3 d < 0.0001, Sham vs. 7 d < 0.0001. *P* value (>0.75); Sham vs. 3 d = 0.0008, Sham vs. 7 d < 0.0001. *n*; Sham = 9, 3 d = 6, 7 d = 8. Data are presented as the mean ± SEM; ***P* < 0.01, ****P* < 0.001, *****P* < 0.0001; two-way ANOVA with Dunnett's multiple comparison test.

 © The Author(s)

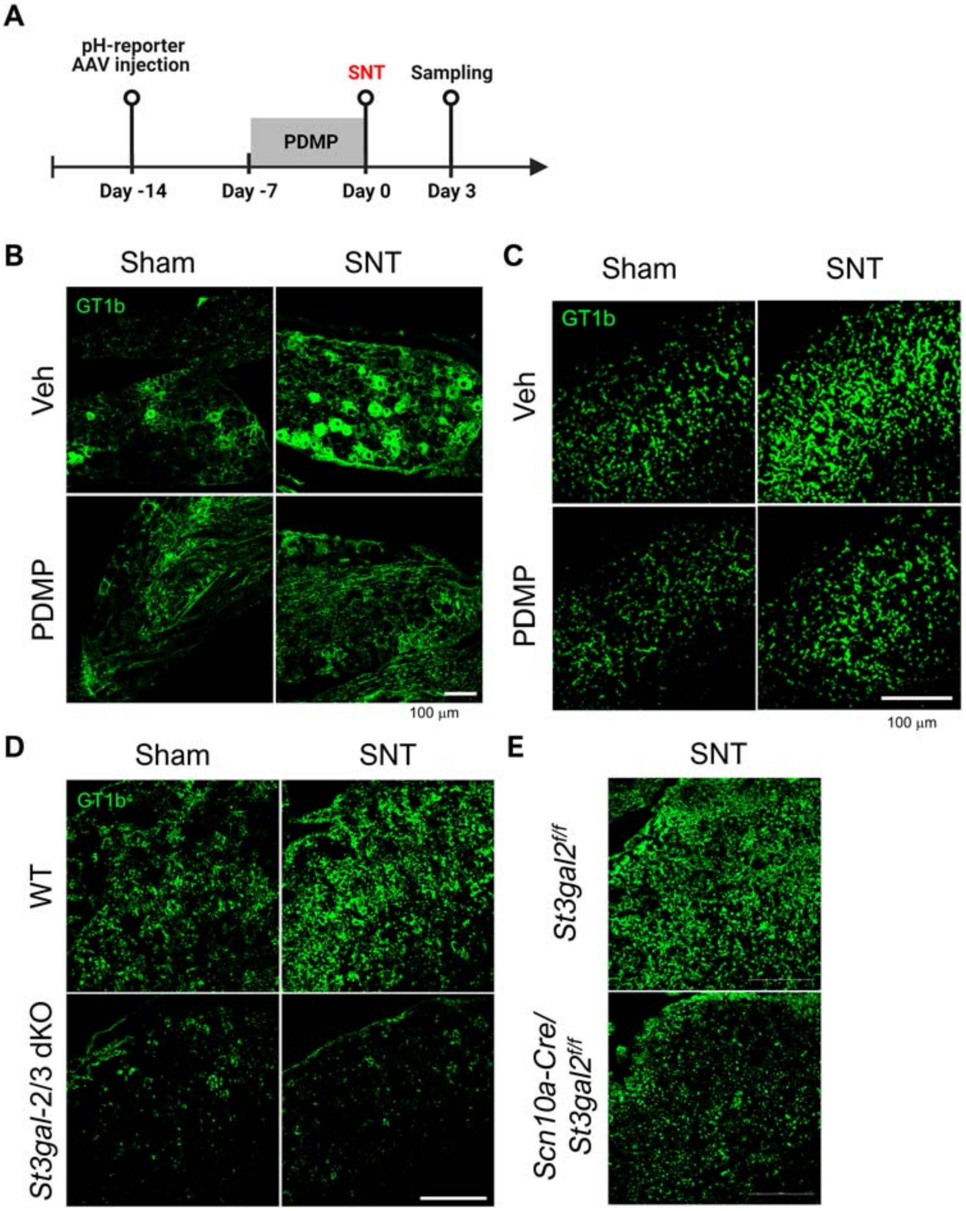


**Figure EV7. Pharmacological and genetic suppression of GT1b synthesis.**

(**A**) Experimental timeline for in vivo inhibition of GT1b synthesis using PDMP. (**B**) Representative immunostaining for GT1b in the DRG and (**C**) spinal cord following nerve injury. (**D**) Immunostaining of spinal cords from *St3gal2/3* knockout and (**E**) *Scn10a-Cre/St3gal2$^{f/f}$* mice with GT1b. Scale bar = 100 µm.

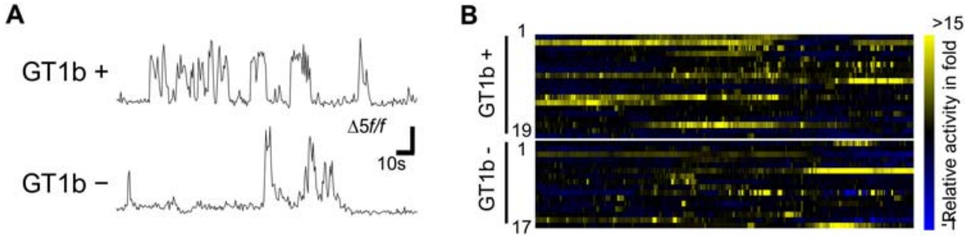


**Figure EV8. Representative trace and heatmap of sCaTs in cultured DRG neurons.**

(**A**) Representative trace and (**B**) heatmap of $Ca^{2+}$ activity.

 © The Author(s)

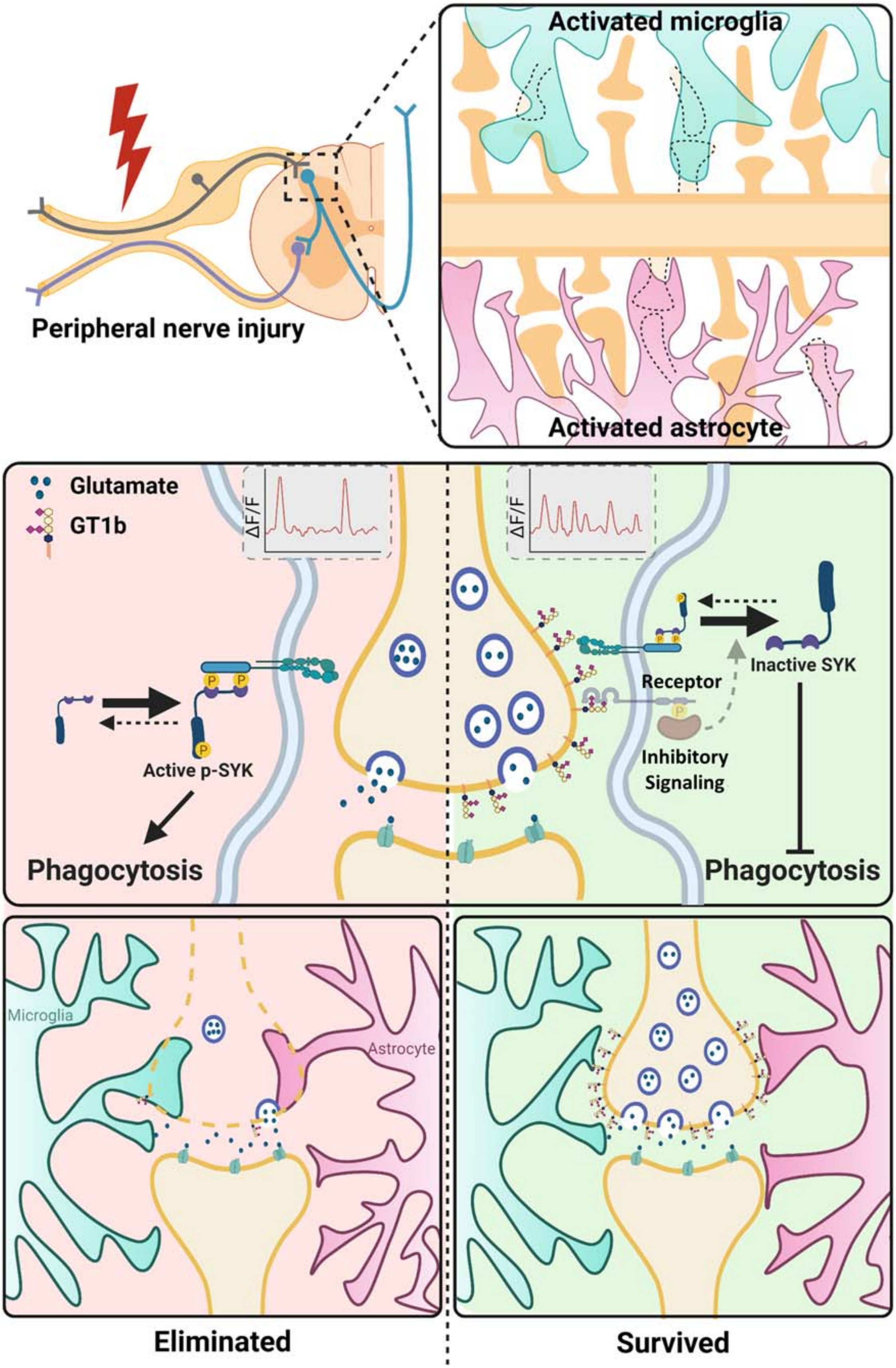
Peripheral nerve injury
Activated microglia
Activated astrocyte
Glutamate
GT1b
ΔF/F
ΔF/F
Active p-SYK
Phagocytosis
Receptor
Inactive SYK
Inhibitory
Signaling
Phagocytosis
Microglia
Astrocyte
Eliminated
Survived

**Figure EV9. Graphical summary.**

This diagram illustrates the proposed mechanism whereby GT1b acts as a "don't eat me" signal to inhibit glial phagocytosis, preventing the elimination of synapses during spinal synapse remodeling after nerve injury. It shows activated microglia and astrocytes in the vicinity of a synapse and how the presence of GT1b on active synapses can protect them from glial phagocytosis.

 © The Author(s)
